# Integrated Management of Cardiovascular–Renal–Hepatic–Metabolic Syndrome: Expanding Roles of SGLT2is, GLP-1RAs, and GIP/GLP-1RAs

**DOI:** 10.3390/biomedicines13010135

**Published:** 2025-01-08

**Authors:** Nikolaos Theodorakis, Maria Nikolaou

**Affiliations:** 1 NT-CardioMetabolics, Clinic for Metabolism and Athletic Performance, 47 Tirteou Str., 17564 Palaio Faliro, Greece; 2 Department of Cardiology & Preventive Cardiology Outpatient Clinic, Amalia Fleming General Hospital, 14, 25th Martiou Str., 15127 Melissia, Greece; m.nikolaou@flemig-hospital.gr; 3 School of Medicine, National and Kapodistrian University of Athens, 75 Mikras Asias, 11527 Athens, Greece

**Keywords:** cardiometabolic medicine, arterial hypertension, dyslipidemia, diabetes mellitus, obesity, heart failure, chronic kidney disease, metabolic dysfunction-associated steatotic liver disease, cardiovascular–kidney–metabolic syndrome, cardiovascular–renal–hepatic–metabolic syndrome

## Abstract

Cardiovascular–Kidney–Metabolic syndrome, introduced by the American Heart Association in 2023, represents a complex and interconnected spectrum of diseases driven by shared pathophysiological mechanisms. However, this framework notably excludes the liver—an organ fundamental to metabolic regulation. Building on this concept, Cardiovascular–Renal–Hepatic–Metabolic (CRHM) syndrome incorporates the liver’s pivotal role in this interconnected disease spectrum, particularly through its involvement via metabolic dysfunction-associated steatotic liver disease (MASLD). Despite the increasing prevalence of CRHM syndrome, unified management strategies remain insufficiently explored. This review addresses the following critical question: How can novel anti-diabetic agents, including sodium–glucose cotransporter-2 inhibitors (SGLT2is), glucagon-like peptide-1 receptor agonists (GLP-1RAs), and dual gastric inhibitory polypeptide (GIP)/GLP-1RA, offer an integrated approach to managing CRHM syndrome beyond the boundaries of traditional specialties? By synthesizing evidence from landmark clinical trials, we highlight the paradigm-shifting potential of these therapies. SGLT2is, such as dapagliflozin and empagliflozin, have emerged as cornerstone guideline-directed treatments for heart failure (HF) and chronic kidney disease (CKD), providing benefits that extend beyond glycemic control and are independent of diabetes status. GLP-1RAs, e.g., semaglutide, have transformed obesity management by enabling weight reductions exceeding 15% and improving outcomes in atherosclerotic cardiovascular disease (ASCVD), diabetic CKD, HF, and MASLD. Additionally, tirzepatide, a dual GIP/GLP-1RA, enables unprecedented weight loss (>20%), reduces diabetes risk by over 90%, and improves outcomes in HF with preserved ejection fraction (HFpEF), MASLD, and obstructive sleep apnea. By moving beyond the traditional organ-specific approach, we propose a unified framework that integrates these agents into holistic management strategies for CRHM syndrome. This paradigm shift moves away from fragmented, organ-centric management toward a more unified approach, fostering collaboration across specialties and marking progress in precision cardiometabolic medicine.

## 1. Introduction

We are currently facing a pandemic of cardiometabolic diseases, marked by a rising incidence of interconnected conditions such as obesity, diabetes mellitus, arterial hypertension, dyslipidemia, and heart failure (HF) with preserved ejection fraction (HFpEF). This increasing burden has contributed to a plateau in the previously declining cardiovascular disease (CVD) mortality rates. As a result, research has increasingly focused on developing more effective strategies to address cardiometabolic risk factors and established diseases [1].

Over the last five years, the landscape of cardiometabolic medicine has undergone a fundamental transformation. Diseases once managed in isolation—obesity, type 2 diabetes mellitus (T2DM), atherosclerotic CVD (ASCVD), HF, chronic kidney disease (CKD), and metabolic dysfunction-associated steatotic liver disease (MASLD)—are now increasingly recognized as interconnected manifestations of underlying metabolic dysregulation. Collectively, these diseases represent a complex and overlapping spectrum of pathologies. These conditions share common pathophysiological threads, and the new perspective acknowledges that organ systems are not merely affected in parallel but are intricately interconnected. Each organ contributes to a tangled web of chronic inflammation, hormonal imbalance, and metabolic dysregulation that reinforce one another, ultimately accelerating disease progression and leading to poor prognosis and decreased quality of life [2].

**Figure 1:**
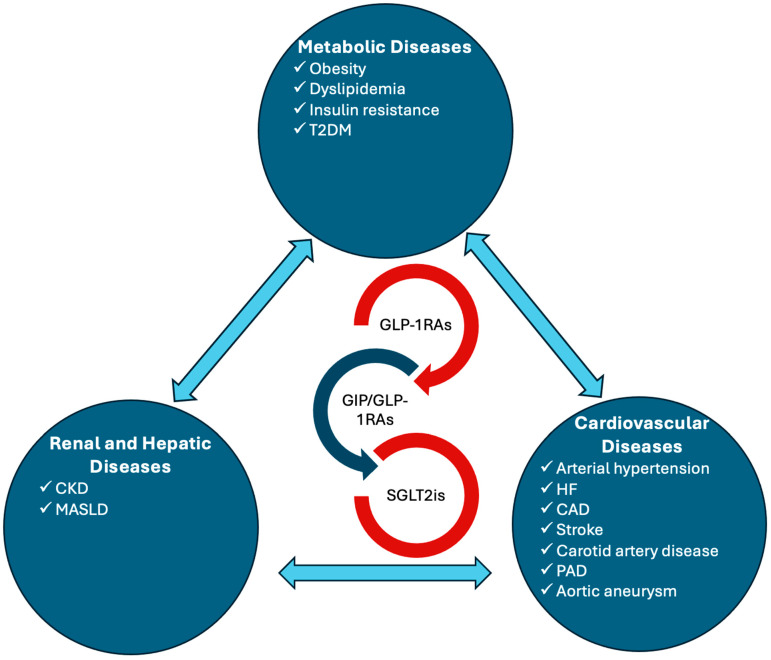
SGLT2is, GLP-1RAs, and GIP/GLP-1RAs for the integrated management of Cardiovascular–Renal–Hepatic–Metabolic syndrome. Abbreviations: CAD (coronary artery disease); CKD (chronic kidney disease); GLP-1RAs (glucagon-like peptide-1 receptor agonists); GIP (Glucose-Dependent Insulinotropic Polypeptide); HF (heart failure); MASLD (metabolic dysfunction-associated steatotic liver disease); PAD (Peripheral Artery Disease); SGLT2is (sodium–glucose cotransporter-2 inhibitors); T2DM (type 2 diabetes mellitus).

Amid this paradigm shift, two major therapeutic classes—sodium–glucose cotransporter-2 inhibitors (SGLT2is) and glucagon-like peptide-1 receptor agonists (GLP-1RAs)—have emerged as true game-changers. Initially developed for glycemic control in T2DM, these agents have far exceeded their original promise. They have demonstrated robust, event-driven benefits, including reducing HF hospitalizations, slowing CKD progression, lowering the rates of major adverse cardiovascular events (MACE), enabling significant and sustained weight loss, and even influencing MASLD pathophysiology and obstructive sleep apnea (OSA) course. Furthermore, the advent of tirzepatide, a dual gastric inhibitory polypeptide (GIP)/GLP-1RA, is raising the bar even higher. These next-generation agents are accelerating weight reduction to levels previously thought unattainable while simultaneously improving multiple cardiometabolic parameters. These wide-ranging benefits have begun to dismantle the rigid boundaries between cardiology, nephrology, endocrinology, and hepatology, fostering integrated treatment strategies instead of fragmented, organ-specific, disease-specific, or specialty-specific approaches (Figure 1) [3,4].

Despite these advances, critical gaps remain in the current literature. Much of the existing medical education, clinical practice, and research predominantly focuses on individual organ systems or diseases, such as ASCVD, HF, CKD, or MASLD, without adequately addressing their complex interdependence or the need for unified management strategies. This fragmented approach fails to account for the intricate bidirectional relationships between these organ systems, where dysfunction in one often exacerbates pathology in others, leading to a vicious cycle of progressive decline. Addressing these gaps requires a paradigm shift from isolated disease management to integrated care models that leverage these transformative therapies.

This review aims to address these research gaps by synthesizing the latest evidence on SGLT2is, GLP-1RAs, and dual agents like tirzepatide, beyond their traditional role in T2DM management. We strive to highlight their transformative impact on the integrated management of the spectrum of Cardiovascular–Renal–Hepatic–Metabolic (CRHM) diseases, explore how they are reshaping clinical practice and research priorities, and provide novel insights into the integration of these therapies as the cornerstones of a unified, multi-system approach amidst the global cardiometabolic pandemic.

## 2. Defining Cardiovascular–Renal–Hepatic–Metabolic Syndrome

Metabolic syndrome (MetS) is well established and has been known in the medical community for many years now, with insulin resistance being considered the major underlying pathophysiological driver [5,6]. It has been defined by the presence of three or more of the following five features:

1. Waist circumference ≥88 cm for women and ≥102 cm for men (≥80 cm for women and ≥90 cm for men if of Asian ancestry).
2. HDL cholesterol <40 mg/dL for men and <50 mg/dL for women.
3. Triglycerides ≥150 mg/dL.
4. Elevated blood pressure (systolic blood pressure [SBP] ≥130 mm Hg or diastolic blood pressure [DBP] ≥80 mm Hg and/or use of antihypertensive medications).
5. Fasting blood glucose ≥100 mg/dL and/or use of anti-diabetic medications.

In 2023, the AHA defined Cardiovascular–Kidney–Metabolic (CKM) syndrome as “A systemic disorder characterized by pathophysiological interactions among metabolic risk factors, CKD, and the cardiovascular system leading to multiorgan dysfunction and a high rate of adverse cardiovascular outcomes. CKM syndrome includes both individuals at risk for CVD due to the presence of metabolic risk factors, CKD, or both and individuals with existing CVD that is potentially related to or complicates metabolic risk factors or CKD”. This syndrome has five stages [5,6]:

1. Stage 0: No CKM risk factors.
2. Stage 1: Excess or dysfunctional adiposity.
3. Stage 2: Metabolic risk factors (hypertriglyceridemia, hypertension, MetS, diabetes) or moderate- to high-risk CKD.
4. Stage 3: Subclinical CVD (atherosclerosis, stage B HF) or very-high-risk CKD.
5. Stage 4: Clinical ASCVD or HF.
  I. Stage 4a: Without kidney failure.
  II. Stage 4b: With kidney failure.

To elaborate on this framework, we introduce the term “Cardiovascular–Renal–Hepatic–Metabolic (CRHM) syndrome” as an extension of CKM syndrome by incorporating the liver as a central player in the interconnected pathophysiology of these organ systems. While CKM syndrome primarily focuses on the interplay between cardiovascular, renal, and metabolic dysfunction, CRHM disease acknowledges the liver’s critical role in these pathophysiological interactions. A pivotal contributor within this framework is MASLD, which represents the hepatic manifestation of systemic metabolic dysregulation and its progression to metabolic dysfunction-associated steatohepatitis (MASH). MASLD is bidirectionally associated with insulin resistance, dyslipidemia, and systemic chronic inflammation, and as a result, it can further exacerbate the progression of atherosclerosis, HF, and CKD. This cascade creates a synergistic progression of disease, with MASLD functioning as both a driver and a consequence of the metabolic and inflammatory imbalances seen in CRHM syndrome. Based on this syndrome, the proposed modified staging could be the following:

1. Stage 0: No CRHM risk factors.
2. Stage 1: Excess or dysfunctional adiposity.
3. Stage 2: Metabolic risk factors (hypertriglyceridemia, hypertension, MetS, diabetes), or moderate- to high-risk CKD, or MASLD with grade 0/1 fibrosis.
4. Stage 3: Subclinical CVD (atherosclerosis, stage B HF), or very-high-risk CKD, or MASLD with grade ≥2 fibrosis.
5. Stage 4: Clinical ASCVD or HF.
  I. Stage 4a: Without kidney failure or liver failure.
  II. Stage 4b: With kidney failure and/or liver failure.

**Figure 2:**
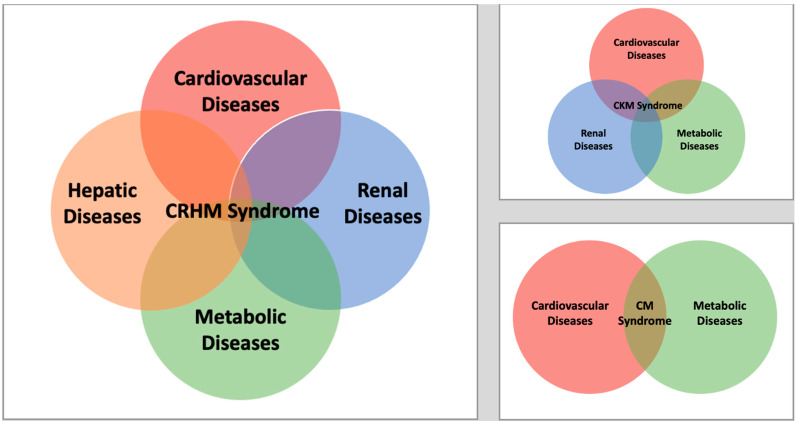
The spectrum of Cardiovascular–Renal–Hepatic–Metabolic diseases and its distinction from other frameworks. The spectrum of cardiometabolic diseases encompassing the cardiometabolic (CM), Cardiovascular–Kidney–Metabolic (CKM), and Cardiovascular–Renal–Hepatic–Metabolic (CRHM) frameworks. This figure illustrates the progression from isolated cardiometabolic interactions to the broader integration of renal and hepatic contributions. The CRHM framework uniquely emphasizes the liver’s role in systemic inflammation, insulin resistance, and lipid dysregulation, with metabolic dysfunction-associated steatotic liver disease serving as a central component. This comprehensive model highlights the interconnected pathophysiology across the cardiovascular, renal, hepatic, and metabolic systems, differentiating CRHM from the narrower CKM and CM spectrums.

Figure 2 illustrates the unique role of hepatic dysfunction, including MASLD, in CRHM syndrome compared to CKM syndrome and cardiometabolic (CM) syndrome.

## 3. Materials and Methods

A comprehensive literature review was conducted to identify relevant studies published up to 31 December 2024. The search was performed using electronic databases, including PubMed and Embase. The search strategy incorporated both free-text terms and controlled vocabulary, such as Medical Subject Headings (MeSH), to ensure the thorough coverage of the relevant literature. Boolean operators (AND, OR) were applied to systematically combine search terms and refine the search process. Key terms included “Sodium-Glucose Cotransporter-2 Inhibitors”, “SGLT2 inhibitors”, “Glucagon-Like Peptide-1 Receptor Agonists”, “GLP-1 receptor agonists”, “dual GIP/GLP-1 receptor agonists”, “Tirzepatide”, “cardiometabolic diseases”, “heart failure”, “chronic kidney disease”, “metabolic-associated steatotic liver disease”, “obesity”, “diabetes mellitus”, “cardiovascular disease”, and “coronary artery disease”. The searches were not restricted by publication type but were limited to studies written in English. Additionally, the reference lists of all retrieved articles were manually reviewed to identify further relevant studies that were not captured by the initial database search. Any discrepancies in eligibility assessment were resolved through discussion among investigators, ensuring consensus in the selection process.

## 4. Mechanistic Insights: Pathophysiological Pathways and Molecular Interventions

The cardioprotective effects of SGLT2is and GLP-1RAs arise from the convergence of molecular mechanisms that go well beyond glucose lowering. One central aspect is the enhancement in myocardial energetics. Under the conditions of metabolic derangement, the heart often relies excessively on fatty acid oxidation, leading to lipotoxicity and mitochondrial dysfunction. SGLT2is enhance ketone body utilization, and GLP-1RAs enhance glucose utilization. As a result, both classes of medications shift myocardial substrate utilization toward more efficient pathways and decrease harmful lipid intermediates and reactive oxygen species. This metabolic shift preserves mitochondrial integrity, improves ATP yield, and mitigates cellular injury [7,8]. In parallel, they influence the key regulators of myocardial structure—including transforming growth factor-β, connective tissue growth factor, and matrix metalloproteinases—thereby reducing fibrosis, limiting myocardial stiffening, and facilitating a healthier balance between collagen deposition and degradation. The net effect is enhanced contractile performance, improved diastolic relaxation, and more balanced cardiac hemodynamics [3,9].

Beyond the myocardium, these drugs impart systemic vascular benefits. GLP-1 receptor activation, through G protein-coupled receptor signaling and increased cAMP, boosts nitric oxide (NO) production and maintains NO bioavailability [10]. Meanwhile, SGLT2is confer endothelial protection by decreasing oxidative stress, fostering antioxidant enzyme activity, and attenuating endothelial cell apoptosis [11]. Both drug classes inhibit pathways leading to endothelial dysfunction, reduce arterial stiffness, and improve coronary flow reserve. They blunt vasoconstrictor stimuli from the renin–angiotensin–aldosterone system and sympathetic nervous system, diminishing neurohormonal activation and lowering angiotensin II and aldosterone levels. As a result, systemic vasodilation and reduced afterload improve stroke volume and cardiac output, while moderated ventricular filling pressures ease the burden on the heart, averting the vicious cycle of volume overload and adverse remodeling [10,11].

At the inflammatory and immunometabolic interface, these agents modulate a spectrum of cytokines, chemokines, and adipokines. SGLT2is, for instance, reduce the infiltration of pro-inflammatory macrophages in adipose tissue and the myocardium, lowering the levels of interleukin-6, tumor necrosis factor-α, and other circulating mediators that propagate endothelial injury and plaque instability. GLP-1RAs similarly downregulate inflammatory signaling and shift adipokine profiles toward protective adiponectin while limiting the secretion of detrimental leptin and resistin. By maintaining a more anti-inflammatory milieu, they stabilize atherosclerotic lesions, protect against ischemic injury, and preserve the electrophysiological stability of the heart, decreasing the likelihood of arrhythmogenic substrates and sudden cardiac events [12,13,14].

On a more granular molecular level, these therapies engage intracellular signaling cascades that govern cellular metabolism and survival. Both SGLT2is and GLP-1RAs can influence AMP-activated protein kinase (AMPK), a critical energy sensor that promotes glucose uptake, fatty acid oxidation, and autophagy while restraining lipogenesis. By activating AMPK and modulating peroxisome proliferator-activated receptor isoforms, they enhance metabolic flexibility and support the maintenance of cardiomyocyte homeostasis. Additional transcriptional regulators—such as Sirtuins and Forkhead box O proteins—are influenced as well, improving cellular resilience against oxidative stress and preserving telomere integrity. The downstream effects consist of greater cardiomyocyte survival, enhanced regenerative signaling, and improved vascular smooth muscle cell function, culminating in a robust defense against progression to HF and vascular disease [15,16].

Furthermore, both drug classes impact ionic homeostasis within cardiomyocytes. SGLT2is may indirectly reduce sodium–hydrogen exchanger activity and intracellular sodium concentration, thus curbing calcium overload and mitigating diastolic dysfunction [17]. GLP-1RAs, by enhancing cAMP-dependent pathways, can improve calcium handling and contractile function [14]. Together, these adjustments in ionic flux, combined with improved metabolic substrate utilization and robust anti-inflammatory, antifibrotic, and endothelium-preserving actions, create a stable cardiovascular (CV) environment. The integrated result is a heart and vascular network that are more energetically efficient, structurally resilient, and biologically adaptable, offering a powerful shield against the multifaceted challenges posed by CRHM syndrome [17,18].

In parallel with their cardiac and vascular actions, SGLT2is and GLP-1RAs confer notable renal benefits that synergize with their systemic hemodynamic improvements. By promoting afferent arteriolar vasodilation and optimizing intraglomerular pressure, these agents help maintain or improve the estimated glomerular filtration rate (eGFR) in the CKD and HF contexts. SGLT2is, in particular, induce a mild, natriuretic diuresis that offloads excess intravascular volume, thereby reducing venous return and left ventricular end-diastolic pressure [3,19]. GLP-1RAs similarly preserve renal perfusion and are associated with favorable effects on tubuloglomerular feedback and inflammatory pathways within the nephron [3,20]. Together, these renal adaptations support euvolemia—an optimal circulatory volume state that alleviates volume overload and improves forward cardiac output. This integrated cardiorenal support not only stabilizes hemodynamics but also lessens the progression of adverse remodeling, lowers ventricular filling pressures, and further solidifies the foundation for improved long-term CV health [3,19,20].

**Figure 3:**
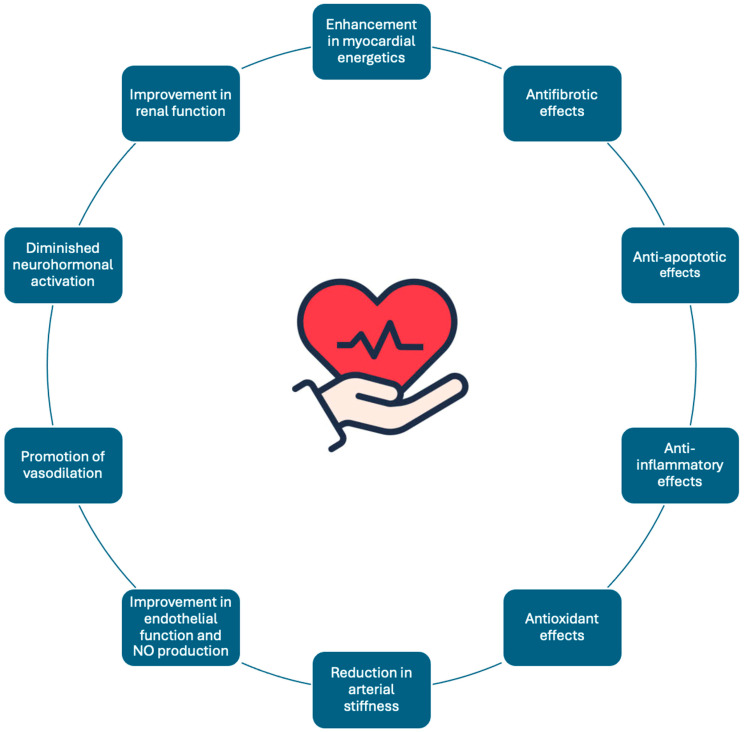
The key cardioprotective mechanisms of GLP-1RAs and SGLT2is.

A summary of the key cardioprotective mechanisms of GLP-1RAs and SGLT2is is illustrated in Figure 3.

## 5. Obesity and OSA

### 5.1. GLP-1RAs

The SCALE Obesity and Prediabetes trial included 3731 adults with obesity (body mass index [BMI] ≥30 kg/m^2^) or overweight (BMI ≥27 kg/m^2^) plus at least one weight-related comorbidity but without T2DM. Participants were randomized to receive once-weekly subcutaneous liraglutide 3 mg or placebo (in a 2:1 ratio) plus lifestyle interventions over 56 weeks. The co-primary endpoints were the percentage change in body weight and the proportion of participants losing at least 5% and more than 10% of their initial body weight. Liraglutide caused a mean weight loss of 8% vs. 2.6% with placebo (difference −5.4%; 95% confidence interval [CI], −5.8% to −5%; *p* < 0.001). Additionally, 63.2% and 33.1% of liraglutide-treated participants achieved ≥5% and ≥10% weight loss compared to 27.1% and 10.6% of those on placebo, respectively (*p* < 0.001 for all comparisons). Improvements were also seen in cardiometabolic risk factors, such as waist circumference, blood pressure, and lipid profiles. Subgroup analyses showed consistent benefits across various baseline BMIs. Regarding safety, liraglutide was associated with gastrointestinal side effects which were mild in 94% of cases. A total of 6.4% of liraglutide-treated patients discontinued due to these events compared to 0.7% in the placebo group. Gallbladder-related events occurred in 2.5% of liraglutide patients versus 1.0% in placebo, while pancreatitis was confirmed in 0.4% of liraglutide patients compared to <0.1% in placebo. The incidence of neoplasms was similar between groups, with 1.9 events per 100 patient-years in the liraglutide group versus 2.4 events per 100 patient-years in the placebo group. These findings established liraglutide 3 mg once weekly as a safe and effective anti-obesity therapy, significantly outperforming lifestyle interventions alone [21].

The SCALE Diabetes trial included 846 adults with BMI ≥27 kg/m^2^ and T2DM. Participants were randomized to receive once-weekly subcutaneous liraglutide 3 mg, liraglutide 1.8 mg (the approved dose for diabetes), or placebo (in a 2:1:1 ratio) plus lifestyle interventions over 56 weeks. The co-primary endpoints were the percentage change in body weight and the proportion of participants losing at least 5% and more than 10% of their initial body weight. Weight loss was 6.0% with liraglutide at a 3.0 mg dose, 4.7% with liraglutide at a 1.8 mg dose, and 2.0% with placebo. The estimated difference in weight loss was −4.00% (95% CI: −5.10% to −2.90%) for liraglutide 3.0 mg compared to placebo and −2.71% (95% CI: −4.00% to −1.42%) for liraglutide 1.8 mg compared to placebo, with both differences being statistically significant (*p* < 0.001). A weight loss of 5% or more was achieved by 54.3% of participants receiving liraglutide 3.0 mg and 40.4% of those receiving liraglutide 1.8 mg, compared to 21.4% with placebo. A weight loss exceeding 10% occurred in 25.2% of participants treated with liraglutide 3.0 mg and 15.9% of those on liraglutide 1.8 mg, compared to 6.7% with placebo. Additionally, liraglutide-treated patients demonstrated greater reductions in HbA1c (−1.3% with liraglutide 3 mg and −1.1% with liraglutide 1.8 mg vs. −0.3% with placebo; *p* < 0.001). Improvements were also seen in cardiometabolic risk factors, such as waist circumference, blood pressure, and lipid profiles. Most adverse events were mild in severity (78% with liraglutide 3 mg, 74% with liraglutide 1.8 mg, 69% with placebo). This study was not designed with sufficient statistical power to draw definitive conclusions regarding safety. These data reinforced liraglutide as a dual-impact therapy for both significant weight reduction and improved metabolic control in patients with T2DM [22].

The STEP 1 trial included 1961 adults with obesity (BMI ≥30 kg/m^2^) or overweight (BMI ≥27 kg/m^2^) plus at least one weight-related comorbidity but without T2DM. Participants were randomized to receive once-weekly subcutaneous semaglutide 2.4 mg or placebo (in a 2:1 ratio) plus lifestyle interventions over 68 weeks. The co-primary endpoints were the percentage change in body weight and the proportion of participants achieving at least a 5% weight reduction. Semaglutide caused a mean weight loss of 14.9% vs. 2.4% with placebo (difference ~12.4%; 95% confidence interval [CI], −13.4% to −11.5%; *p* < 0.001). Additionally, 86.4%, 69.1%, and 50.5% of semaglutide-treated participants achieved ≥5%, ≥10%, and ≥15% weight loss compared to 31.5%, 12%, and 4.9% of those on placebo, respectively (*p* < 0.001 for all comparisons). Lean mass loss constitutes roughly 35% of the total weight loss, while semaglutide also reduces the fat-to-lean-mass ratio. Improvements were also seen in cardiometabolic risk factors, such as waist circumference, blood pressure, and lipid profiles. Subgroup analyses showed consistent benefits across various demographics and baseline BMIs. Regarding safety, gastrointestinal disorders were the most commonly reported adverse events, occurring more frequently in participants treated with semaglutide compared to those receiving placebo (74.2% vs. 47.9%). These events were generally mild to moderate in severity, temporary, and typically resolved without the need for the permanent discontinuation of treatment. However, treatment discontinuation due to adverse events, primarily gastrointestinal, was higher in the semaglutide group than in the placebo group (7.0% vs. 3.1%). Gallbladder-related conditions, predominantly cholelithiasis, were reported in 2.6% of semaglutide-treated participants compared to 1.2% in the placebo group. Mild acute pancreatitis, classified using the Atlanta criteria, was observed in three participants in the semaglutide group. No differences were noted between the groups regarding the incidence of benign or malignant neoplasms. These findings established semaglutide 2.4 mg once weekly as a safe and effective anti-obesity therapy, with even more pronounced weight loss effects compared to liraglutide [23].

In an extension of the STEP 1 trial, 327 participants were followed up with following the discontinuation of semaglutide and lifestyle interventions from week 68 to week 120. By week 120, weight regain averaged 11.6% in the semaglutide group and 1.9% in the placebo group. This resulted in overall weight changes from week 0 to week 120 of a 5.6% loss for semaglutide and a 0.1% loss for placebo. Furthermore, cardiometabolic benefits observed with semaglutide up to week 68 largely reverted towards baseline by week 120 [24].

The STEP 5 trial followed a similar design to the STEP 1 trial but included a two-year follow-up period and a small sample (*n* = 304 patients). The average change in body weight from baseline to week 104 was −15.2% in the semaglutide 2.4 mg group compared to −2.6% in the placebo group, corresponding to an estimated treatment difference of −12.6% (95% CI, −15.3% to −9.8%; *p* < 0.0001). Furthermore, a greater proportion of participants in the semaglutide group achieved at least 5% weight loss from baseline in week 104 compared to the placebo group (77.1% versus 34.4%; *p* < 0.0001) [25].

The STEP 2 trial included 1210 adults with T2DM and overweight or obesity, randomized to once-weekly subcutaneous semaglutide 2.4 mg, semaglutide 1.0 mg (the approved dose for diabetes), or placebo (in a 1:1:1 ratio) plus lifestyle interventions for 68 weeks. The co-primary endpoints were the percentage change in body weight and the proportion of participants achieving at least a 5% weight reduction. Weight loss was 9.6% with semaglutide at a 2.4 mg dose, 7.0% with semaglutide at a 1.0 mg dose, and 3.4% with placebo. The estimated difference in weight loss was −6.2% (95% CI: −7.3% to −5.2%) for semaglutide 2.4 mg compared to placebo and −3.6% (95% CI: −4.7% to −2.5%) for semaglutide 1.0 mg compared to placebo, with both differences being statistically significant (*p* < 0.001). A weight loss of 5% or more was achieved by 68.8% of participants receiving semaglutide 2.4 mg and 57.1% of those receiving semaglutide 1.0 mg, compared to 28.5% with placebo. Additionally, semaglutide-treated patients demonstrated greater reductions in HbA1c (−1.6% with semaglutide 2.4 mg and −1.1% with semaglutide 1.0 mg vs. −0.4% with placebo; *p* < 0.001). Subgroup analyses showed consistent benefits regardless of baseline BMI or glycemic control status. Gastrointestinal adverse events, primarily mild to moderate in severity, were reported in 63.5% of patients receiving semaglutide 2.4 mg, 57.5% of patients receiving semaglutide 1.0 mg, and 34.3% of patients receiving placebo. These data reinforced semaglutide 2.4 mg once weekly as a dual-impact therapy for both significant weight reduction and improved metabolic control in patients with T2DM [26].

### 5.2. GIP/GLP-1RAs

Tirzepatide is a novel dual GIP/GLP-1RA that has revolutionized obesity management. The SURMOUNT-1 trial included 2539 adults with obesity (BMI ≥30 kg/m^2^) or overweight (BMI ≥27 kg/m^2^) plus weight-related complications but without diabetes. Patients received once-weekly subcutaneous tirzepatide (5, 10, or 15 mg) or placebo (in a 1:1:1:1 ratio), alongside lifestyle interventions, for 72 weeks. The co-primary endpoints were the percentage change in body weight and the proportion of participants achieving at least a 5% weight reduction. Tirzepatide 15 mg enabled a 20.9% mean weight reduction (95% CI, −21.8% to −19.9%) vs. 3.1% with placebo (*p* < 0.001), far surpassing conventional therapies. Key secondary endpoints included the proportion of participants achieving ≥20% weight loss was 57% with tirzepatide 15 mg (95% CI, 53% to 61%), compared to 3% with placebo (*p* < 0.001). Subgroup analyses showed consistent weight reduction across age, sex, and baseline BMI categories. Participants in the tirzepatide group also exhibited improvements in blood pressure and lipid profiles (e.g., low-density lipoprotein levels decreasing by approximately 6 mg/dL). Regarding safety, gastrointestinal events were the most frequently reported adverse effects with tirzepatide, typically mild to moderate in severity and occurring mainly during dose escalation. Treatment discontinuation due to adverse events was observed in 4.3%, 7.1%, and 6.2% of participants receiving tirzepatide at doses of 5 mg, 10 mg, and 15 mg, respectively, compared to 2.6% in the placebo group. Four cases of non-severe pancreatitis were reported, distributed evenly across all treatment groups, including the placebo group. The incidence of cholelithiasis was comparable between the tirzepatide and placebo groups. However, cholecystitis and acute cholecystitis were reported more frequently in the tirzepatide groups than in the placebo group, though the overall incidences were low (≤0.6%). SURMOUNT-1 established tirzepatide as a highly efficacious anti-obesity agent, setting new benchmarks in weight management [27].

A recently published extension of SURMOUNT-1 further underscored tirzepatide’s long-term benefits. In participants with pre-diabetes at baseline, three years of tirzepatide 15 mg reduced the risk of progression to T2DM by 93% compared to placebo (HR: 0.07; 95% CI, 0.00% to 0.1; *p* < 0.001), corresponding to an ARR of 12.4% (13.7% incidence with placebo versus 1.3% with tirzepatide). This translates to the prevention of one case of T2DM for every eight individuals over 3 years. Regarding weight reduction at 176 weeks, tirzepatide 15 mg enabled an impressive 22.9% weight reduction (per-protocol analysis) and a 19.7% weight reduction (intention-to-treat analysis). Remarkably, even after a 17-week off-treatment period (at 193 weeks), patients maintained a −17.3% weight reduction from baseline. These extended findings not only reinforce tirzepatide’s potent and durable anti-obesity effects but also highlight its potential to profoundly modify the metabolic trajectory, preventing or delaying the onset of diabetes in high-risk individuals. SURMOUNT-1, along with its extension, thus established tirzepatide as a highly efficacious and potentially disease-modifying therapy in obesity and pre-diabetes, setting new benchmarks in long-term weight management and metabolic risk reduction [28].

The SURMOUNT-2 trial recruited about 938 adults with T2DM and overweight or obesity to receive once-weekly subcutaneous tirzepatide (5, 10, or 15 mg) or placebo (in a 1:1:1:1 ratio), alongside lifestyle interventions, for 72 weeks. The co-primary endpoints were the percentage change in body weight and the proportion of participants achieving at least a 5% weight reduction. The least-squares mean reduction in body weight at week 72 was –14.7% (SE 0.5) with tirzepatide 15 mg, compared to –3.2% (SE 0.5) with placebo. This corresponds to estimated treatment differences of –11.6% points (95% CI: –13.0 to –10.1) for tirzepatide 15 mg (*p* < 0.0001). A greater proportion of participants treated with tirzepatide achieved body weight reductions of 5% or more compared to those receiving a placebo (79–83% vs. 32%). Regarding glycemic control, tirzepatide showed a mean HbA1c reduction of about −2.1% vs. −0.5% in placebo (*p* < 0.001) [29].

A recent scoping review assessed the potential role of GLP-1RAs in managing OSA and associated hypopnea syndromes. The review included nine studies, comprising randomized controlled trials (RCTs), observational studies, and case reports, investigating the impact of GLP-1RAs on the apnea–hypopnea index (AHI) and related outcomes. Some studies demonstrated significant improvements; for example, an RCT reported a mean AHI reduction of −12.2 events/hour with liraglutide compared to −6.1 events/hour in the placebo group (*p* = 0.015). Another study combining liraglutide with positive airway pressure (PAP) therapy showed a greater reduction in the AHI (26.1 ± 7.1 events/hour) than PAP alone (31.6 ± 6.9 events/hour, *p* < 0.05). However, other studies reported mixed results, with one showing no significant correlation between AHI reduction and weight loss in participants with a BMI >30 kg/m^2^. Despite these promising findings, the evidence is limited by small sample sizes, short follow-up durations (ranging from 4 weeks to 6 months), and varying methodological quality. While GLP-1RAs may offer potential benefits as adjuncts to standard therapies, further rigorous trials with larger populations and longer follow-up are needed to establish their efficacy in OSA management [30].

The SURMOUNT-OSA trial enrolled adults with obesity and moderate-to-severe OSA without diabetes to evaluate the effect of once-weekly subcutaneous tirzepatide on the AHI and related cardiometabolic factors over 52 weeks. The study consisted of two trials: the first included 234 patients under positive airway pressure (PAP) therapy, while the second enrolled 235 patients also using PAP. The primary endpoint for both trials was the change in the AHI from baseline. The use of tirzepatide (10–15 mg) led to a significant reduction in the AHI (approximately –20 events/hour compared to placebo; *p* < 0.001) in both trials, demonstrating a clinically meaningful improvement in OSA severity irrespective of PAP use. Additionally, improvements in sleep-related quality of life measures were observed, highlighting the potential of tirzepatide’s robust metabolic effects to address OSA and reshape the management of this obesity-related comorbidity [31].

### 5.3. Key Considerations for the Use of GLP-1RAs and GIP/GLP-1RAs in Obesity

The findings from trials investigating GLP-1RAs and dual GIP/GLP-1RAs in obesity and OSA represent a significant advancement in managing these conditions, yet there are critical aspects to analyze. The STEP and SURMOUNT trials have firmly established these agents as highly effective interventions, enabling profound weight reductions and improvements in cardiometabolic health markers. Semaglutide’s ability to enable mean weight losses of nearly 15% in patients without T2DM and 10% in those with T2DM, alongside improvements in glycemic control and other CV risk factors, underscores its transformative potential in obesity management.

Similarly, the use of tirzepatide has led to weight reductions exceeding 20% in patients without type 2 diabetes (T2DM) and over 10% in those with T2DM. Additionally, its ability to delay the progression to T2DM and significantly improve glycemic control in patients with T2DM highlights its profound impact on the prevention and management of metabolic diseases. However, the reasons why semaglutide and tirzepatide lead to lower rates of body weight reduction—up to 50% less—in patients with T2DM compared to those without T2DM have not been fully elucidated.

Notably, findings from an extension of the STEP 1 trial revealed that discontinuing semaglutide leads to the regain of approximately two-thirds of the lost weight within one year, emphasizing the importance of maintaining continuous treatment to sustain weight loss benefits.

However, an important practical limitation warrants careful consideration. The highly controlled nature of these trials, which incorporated lifestyle interventions and closely monitored participants, raises questions about the generalizability of these results to real-world clinical settings. Adherence to treatment regimens and accessibility to these high-cost therapies may pose significant challenges outside the structured environments of clinical trials. Furthermore, the long-term safety profiles of these agents remain uncertain and necessitate evaluation through prospective cohort studies or case–control studies to identify potential adverse effects and ensure their sustained benefit.

Looking forward, the limitations of these studies underscore the need for long-term real-world data to evaluate the durability of weight loss benefits and long-term safety. Additionally, mechanistic studies are needed to better understand how these agents influence OSA and other obesity-related complications beyond weight loss.

## 6. CVD

### 6.1. Arterial Hypertension

Sodium–glucose cotransporter-2 inhibitors (SGLT2is) have demonstrated significant blood pressure-lowering effects in patients with type 2 diabetes mellitus (T2DM) and hypertension, independent of their glucose-lowering properties. These agents exhibit modest but clinically meaningful reductions in both SBP and DBP. A recent meta-analysis of RCTs showed that SGLT2is reduce 24 h ambulatory SBP by approximately 5.06 mmHg (95% CI: −7.10 to −3.01) and DBP by 2.39 mmHg (95% CI: −4.11 to −0.67). Similarly, office BP measurements demonstrate reductions of 4.53 mmHg in SBP and 2.12 mmHg in DBP [24]. Notably, non-obese patients with T2DM and CKD achieved greater reductions in SBP, approximately 12 mmHg, suggesting that baseline BMI and baseline renal function may influence efficacy. This differential response could be related to variations in sodium retention and sympathetic nervous system activity [32].

The BP-lowering effects of SGLT2is are primarily driven by mechanisms that include osmotic diuresis and natriuresis. By inhibiting sodium and glucose reabsorption in the proximal tubule, these agents enhance natriuresis, leading to reduced plasma volume and preload. The long-term use of SGLT2is has also been linked to decreased sympathetic nerve activity, possibly mediated through central nervous system pathways such as the rostral ventrolateral medulla and nucleus tractus solitarius. In addition, evidence suggests that SGLT2is may improve arterial stiffness and vascular compliance, though this requires further exploration [33].

A meta-analysis examining the impact of GLP-1RAs on blood pressure in individuals with and without type 2 diabetes revealed modest reductions in SBP compared to placebo. Specific findings included a mean difference (MD) of −3.40 mmHg (95% CI: −4.22 to −2.59; *p* < 0.001) for semaglutide, −2.61 mmHg (95% CI: −3.48 to −1.74; *p* < 0.001) for liraglutide, −1.46 mmHg (95% CI: −2.20 to −0.72; *p* < 0.001) for dulaglutide, and −3.36 mmHg (95% CI: −3.63 to −3.10; *p* < 0.001) for exenatide. The magnitude of SBP reduction generally increased with longer treatment durations. DBP reductions were statistically significant only with exenatide (MD: −0.94 mmHg; 95% CI: −1.78 to −0.1; *p* = 0.03). For semaglutide, reductions in SBP were closely linked to decreases in glycated hemoglobin levels and BMI. This indicates that the blood pressure-lowering effects of GLP-1RAs are likely indirect effects [34].

A post hoc analysis of the SURMOUNT-1 trial demonstrated that tirzepatide significantly reduced SBP by 6.8 mm Hg (95% CI: −7.4 to −6.2; *p* < 0.001) and DBP by 4.2 mm Hg (95% CI: −4.6 to −3.8; *p* < 0.001) compared to placebo over 72 weeks. The BP reduction occurred rapidly within the first 24 weeks of treatment, after which values stabilized until the study’s end. Notably, 58.0% of participants treated with tirzepatide achieved normal blood pressure (SBP <130 mm Hg and DBP <80 mm Hg) at week 72, compared to 35.2% in the placebo group (*p* < 0.001). The BP-lowering effects were consistent across baseline BP subgroups and hypertension categories, as demonstrated by a shift in the BP distribution curve toward lower values. Mediation analysis indicated that weight loss explained 68% of the SBP reduction and 71% of the DBP reduction (*p* < 0.001 for both). While low BP-related adverse events were rare, they were slightly more common in the tirzepatide group compared to placebo. These results highlight the substantial and clinically meaningful BP improvements achieved with tirzepatide, primarily driven by its potent weight-reducing effects [35].

### 6.2. ASCVD

The first insights into the cardioprotective effects of GLP-1RAs and SGLT2is emerged from trials conducted on diabetic patients.

Seven key trials examined the impact of SGLT2 inhibitors on CV outcomes in patients with diabetes: the EMPA-REG OUTCOME trial, the CANVAS program (analyzing two trials together), the DECLARE-TIMI 58 trial, the CREDENCE trial, the VERTIS CV trial, and the SCORED trial. A meta-analysis of six of these trials included a total of 46,969 participants with T2DM, of whom 66.2% had ASCVD. The findings indicated that SGLT2 inhibitors reduced the risk of MACE (HR, 0.90; 95% CI, 0.85–0.95; Q statistic, *p* = 0.27) and the composite outcome of HF hospitalization or CV death (HR, 0.78; 95% CI, 0.73–0.84; Q statistic, *p* = 0.09). Risk reduction for HF hospitalization was consistently observed across all trials (HR, 0.68; 95% CI, 0.61–0.76; I2 = 0.0%). However, there was significant heterogeneity in the association with CV death (HR, 0.85; 95% CI, 0.78–0.93; Q statistic, *p* = 0.02; I2 = 64.3%). Additionally, the presence or absence of ASCVD did not alter the associations with outcomes for MACE (HR, 0.89; 95% CI, 0.84–0.95 and HR, 0.94; 95% CI, 0.83–1.07, respectively; interaction *p* = 0.63), the composite of HF hospitalization or CV death (interaction *p* = 0.62), or HF hospitalization (interaction *p* = 0.26) [36].

Eight major trials assessed the CV outcomes of GLP-1RAs in patients with T2DM: the ELIXA trial, LEADER trial, SUSTAIN 6 trial, EXSCEL trial, HARMONY Outcomes trial, REWIND trial, PIONEER 6 trial, and AMPLITUDE-O trial. A meta-analysis of these trials, encompassing 60,080 participants (of whom 76.6% had established CVD), found that GLP-1RAs were associated with a 14% reduction in MACE (HR 0.86, 95% CI 0.80–0.93, *p* < 0.0001), with consistent effects across different GLP-1RA structures and subgroups (all *p* interaction ≥0.14). Moreover, GLP-1RAs reduced all-cause mortality by 12% (HR 0.88, 95% CI 0.82–0.94, *p* = 0.0001) and hospitalizations for HF by 11% (HR 0.89, 95% CI 0.82–0.98, *p* = 0.013), without increasing the risks of severe hypoglycemia, retinopathy, or pancreatic complications. Notably, in the seven trials, the point estimate for risk reduction was lower in those with established ASCVD (HR 0.85) compared to those without (HR 0.94), with a borderline interaction *p*-value of 0.068. This finding suggests, though does not conclusively establish, that GLP-1RAs may confer greater benefit in patients with established ASCVD [37].

The results of these meta-analyses provided the rationale for conducting further studies to evaluate the effects of SGLT2is in HF and GLP-1RAs in HF and ASCVD, irrespective of diabetes status.

The SELECT trial enrolled 17,604 adults with overweight or obesity (BMI ≥27 kg/m^2^) and established ASCVD but notably without a diagnosis of T2DM. Participants were assigned to once-weekly subcutaneous semaglutide 2.4 mg or placebo, in addition to standard-of-care therapy, and followed for a median of about five years. The primary endpoint was a three-component MACE composite—time to the first occurrence of CV death, nonfatal myocardial infarction (MI), or nonfatal stroke. Semaglutide 2.4 mg reduced the primary endpoint by 20% compared to placebo (HR: 0.80; 95% CI: 0.72–0.90; *p* < 0.001), with event rates of approximately 6.5% vs. 8%—an ARR of 1.5% and an NNT of 67. This trial positioned semaglutide as the first anti-obesity medication to improve hard CV outcomes in non-diabetic patients with established ASCVD. Subgroup analyses indicated that the CV benefits of semaglutide were consistent across key strata, including age groups, sex, and varying baseline CV risks. By confirming robust MACE reduction in a population without diabetes, SELECT expanded the indications of GLP-1RAs, underscoring that weight-centric metabolic interventions can deliver meaningful CV protection in patients with obesity and established ASCVD [38].

Insights into the long-term weight loss effects of semaglutide in non-diabetic patients were further explored in the SELECT trial. In this study, the intention-to-treat (ITT) analysis, which included all participants regardless of their adherence to the treatment protocol, demonstrated that over the 208-week study period, the mean percentage weight loss was −10.2% in the semaglutide group compared to −1.5% in the placebo group, with a treatment difference of −8.7% (95% CI: −9.42% to −7.88%; *p* < 0.0001). The pattern of change in waist circumference (WC) closely followed the trend observed in body weight changes. By week 208, the average reduction in WC was −7.7 cm with semaglutide compared to −1.3 cm with placebo, resulting in a treatment difference of −6.4 cm (95% CI −7.18 to −5.61; P < 0.0001). The per-protocol (PP) analysis, which assessed only participants who remained on treatment, showed a mean weight loss of −11.7% in the semaglutide group versus −1.5% in the placebo group, resulting in a treatment difference of −10.2% (95% CI: −11.0 to −9.42; *p* < 0.0001) [39].

### 6.3. HF

#### 6.3.1. SGLT2is in Chronic HF

The DAPA-HF trial enrolled 4744 patients with symptomatic HF with reduced ejection fraction (HFrEF) and New York Heart Association (NYHA) class II–IV symptoms. The median age was approximately 66 years, and about 21% were female. Roughly 40% had T2DM, while the remainder did not, reflecting a real-world mix. Most patients were receiving background guideline-directed medical therapy (GDMT), including beta-blockers (~96%), renin–angiotensin system inhibitors (RASIs) (~99%), and mineralocorticoid receptor antagonists (~71%). Over a median follow-up of 18.2 months, they received dapagliflozin 10 mg once daily or placebo. The primary endpoint—a composite of worsening HF (hospitalization or urgent intravenous therapy) or CV death—was reduced by 26% (hazard ratio—HR: 0.74; 95% confidence interval—CI: 0.65–0.85; *p* < 0.001), with event rates of 16.3% vs. 21.2%. This absolute risk reduction (ARR) of 4.9% corresponded to a number needed to treat (NNT) of ~21 to prevent one primary endpoint event over 18.2 months. HF hospitalizations were reduced by 30% (HR: 0.70; 95% CI: 0.59–0.83), while CV death was reduced by 18% (HR: 0.82; 95% CI: 0.69–0.98). Subgroup analyses were consistent across diabetic and non-diabetic patients (HR in non-diabetic patients: 0.73; 95% CI: 0.60–0.88; HR in diabetic patients: 0.75; 95% CI: 0.63–0.90), age groups, sex, cause of HF, baseline renal function (including those with eGFR as low as 30 mL/min/1.73 m^2^), and geographic regions. These data confirmed dapagliflozin’s role as an essential, broadly applicable addition to the GDMT of HF, independent of diabetes status, ushering in a new era where SGLT2is join the foundational pillars of HF management [40].

The EMPEROR-Reduced trial included 3730 patients with HFrEF (left ventricular ejection fraction—LVEF ≤40%), NYHA class II–IV symptoms, and a median LVEF of around 27%. About 50% had T2DM, and most patients were receiving background GDMT, including beta-blockers (~95%), RASIs (~99%), and mineralocorticoid receptor antagonists (~70%). The median follow-up was 16 months. Empagliflozin 10 mg once daily significantly reduced the primary endpoint (CV death or HF hospitalization) by 25% (HR: 0.75; 95% CI: 0.65–0.86; *p* < 0.001), lowering event rates from 24.7% to 19.4%. This ARR of ~5.3% translates to an NNT of ~19 to prevent one primary endpoint event over 16 months. HF hospitalizations were reduced by 31% (HR: 0.69; 95% CI: 0.59–0.81). Subgroup analyses showed consistent benefit regardless of diabetic status (HR in non-diabetic patients: 0.78; 95% CI: 0.64–0.97; HR in diabetic patients: 0.72; 95% CI: 0.60–0.87), age groups, sex, race, cause of HF, and baseline renal function (including those with eGFR as low as 20 mL/min/1.73 m^2^). Furthermore, the rate of decline in the eGFR was slower in the empagliflozin group than that in the placebo group (–0.55 mL/min/1.73 m^2^ per year vs. –2.28 mL/min/1.73 m^2^ per year), accounting for a between-group difference of 1.73 mL/min/1.73 m^2^ per year (95% CI, 1.10 to 2.37; *p* < 0.001). Notably, empagliflozin also reduced the composite renal outcome by 50% (HR: 0.50; 95% CI: 0.32–0.77). Together with DAPA-HF, EMPEROR-Reduced demonstrated the class effect of SGLT2is in HFrEF, firmly establishing these agents as part of the GDMT [41].

The DELIVER trial enrolled 6263 patients with HF and an LVEF of >40%, capturing a broad spectrum of patients with mildly reduced to preserved EF. The majority of patients (~75%) had NYHA class II symptoms, while ~45% had T2DM. Over a median follow-up of 2.3 years, dapagliflozin 10 mg once daily significantly reduced the primary endpoint—a composite of CV death or worsening HF event (hospitalization or urgent HF visit)—by 18% compared to placebo (HR: 0.82; 95% CI: 0.73–0.92; *p* = 0.001), with event rates of 16.4% vs. 19.5%. This ARR of 3.1% translates to an NNT of ~33 to prevent one primary endpoint event over 2.3 years. HF hospitalizations were reduced by 23% (HR: 0.77; 95% CI: 0.67–0.89). Subgroup analyses confirmed that dapagliflozin’s effects were robust across various baseline characteristics, including age, sex, LVEF ranges within the preserved and mildly reduced spectrum, diabetic and non-diabetic subgroups (HR in non-diabetic patients: 0.81; 95% CI: 0.68–0.96; HR in diabetic patients: 0.83; 95% CI: 0.70–0.97), and varying degrees of renal function (including those with eGFR as low as 25 mL/min/1.73 m^2^). Together with EMPEROR-Preserved, DELIVER firmly established SGLT2is as foundational therapies in patients with HF beyond the reduced LVEF range [42].

Like the DELIVER trial, the EMPEROR-Preserved trial included 5988 patients with HF and an LVEF of >40%. The majority of patients (~81%) had NYHA class II symptoms, while ~49% had T2DM. Over a median of 26.2 months, the primary endpoint—a composite of CV death or HF hospitalization—was significantly reduced by empagliflozin 10 mg once daily, yielding a 21% relative risk reduction compared to placebo (HR: 0.79; 95% CI: 0.69–0.90; *p* < 0.001). Event rates were 13.8% in the empagliflozin group versus 17.1% in placebo, translating into an ARR of 3.3% and an NNT of 30 over 26.2 months. HF hospitalizations were reduced by 29% (HR: 0.71; 95% CI: 0.60–0.83). Subgroup analyses showed that the benefits were consistent across a broad range of baseline characteristics, including age, sex, LVEF ranges within the preserved and mildly reduced spectrum, diabetic and non-diabetic subgroups (HR in non-diabetic patients: 0.78; 95% CI: 0.64–0.95; HR in diabetic patients: 0.79; 95% CI: 0.67–0.94), and varying degrees of renal function (including those with eGFR as low as 20 mL/min/1.73 m^2^). Together with DELIVER, EMPEROR-Preserved demonstrated the class effect of SGLT2is in HFpEF/HF with mildly reduced ejection fraction (HFmrEF), firmly establishing these agents as part of the GDMT of HF across the entire spectrum of LVEF [43].

#### 6.3.2. Acute HF

The SOLOIST-WHF trial investigated the efficacy of sotagliflozin, a dual SGLT2 and SGLT1 inhibitor, in patients with type 2 diabetes and a recent hospitalization for worsening HF. A total of 1222 patients were randomized to receive either sotagliflozin or placebo, with the first dose administered either before discharge (48.8%) or shortly after discharge (52.2%) (median: 2 days). Over a median follow-up of 9.0 months, the primary endpoint—a composite of total CV deaths, hospitalizations for HF, and urgent visits for HF—was significantly lower in the sotagliflozin group compared to placebo (245 vs. 355 events; HR: 0.67; 95% CI: 0.52–0.85; *p* < 0.001). Event rates were 40.3% in the sotagliflozin group versus 57.8% in the placebo group, translating into an ARR of 17.5% and an NNT of 6 over 9 months. Subgroup analyses confirmed consistent benefits across a range of baseline characteristics, including left ventricular ejection fraction and the timing of initiation. While sotagliflozin was generally well tolerated, diarrhea (6.1% vs. 3.4%) and severe hypoglycemia (1.5% vs. 0.3%) were more common with sotagliflozin than placebo. Despite the early termination of the trial due to funding issues, the findings suggest that the early initiation of sotagliflozin in this high-risk population significantly improves clinical outcomes [44].

The EMPULSE trial evaluated the efficacy and safety of empagliflozin in 530 patients hospitalized for acute HF after clinical stabilization. Patients were randomized 1:1 to receive empagliflozin 10 mg once daily or placebo for 90 days. Empagliflozin significantly improved decongestion-related outcomes compared to placebo, including greater reductions in body weight (adjusted mean difference at Day 15: −1.97 kg; 95% CI: −2.86 to −1.08, *p* < 0.0001), NT-proBNP levels (adjusted geometric mean ratio at Day 15: 0.92; 95% CI: 0.86 to 0.98, *p* = 0.010), and clinical congestion scores. These effects were observed as early as Day 15 and sustained through Day 90. Additionally, empagliflozin was associated with higher hematocrit levels, indicating improved intravascular decongestion. Patients with greater weight loss or hematocrit increase experienced better clinical outcomes at 90 days, including improvements in the hierarchical composite endpoint (win ratio: 1.75; 95% CI: 1.37 to 2.23, *p* < 0.0001). These findings highlight empagliflozin’s potential as an effective and safe decongestive therapy in patients hospitalized with acute HF [45].

#### 6.3.3. GLP-1RAs in Chronic HF

The STEP-HFpEF trial enrolled 529 adults with HFpEF (LVEF ≥50%) and obesity (BMI ≥30 kg/m^2^), all on standard background therapies. Over a 52-week treatment period, patients were randomized to once-weekly subcutaneous semaglutide 2.4 mg or placebo. The dual primary endpoint was the change in the Kansas City Cardiomyopathy Questionnaire (KCCQ) Clinical Summary Score from baseline, a measure of HF-related quality of life, and the change in body weight. The hierarchical composite endpoint included that included death from all causes; HF events (defined as adjudicated events of hospitalization for HF or urgent visits in which intravenous therapy was administered); differences of at least 15, at least 10, and at least 5 points in the change in the KCCQ-CSS from baseline; and a difference of at least 30 m in the change in the 6-min walk distance from baseline. The average improvement in the KCCQ-CSS was 16.6 points with semaglutide compared to 8.7 points with placebo, resulting in a difference of 7.8 points (95% CI, 4.8 to 10.9; *p* < 0.001). The mean percentage reduction in body weight was −13.3% with semaglutide versus −2.6% with placebo, with a difference of −10.7% (95% CI, −11.9% to −9.4%; *p* < 0.001). Secondary outcomes demonstrated positive changes in the 6 min walk distance, with a mean increase of 21.5 m for semaglutide compared to 1.2 m for placebo (difference of 20.3 m; 95% CI, 8.6 to 32.1; *p* < 0.001). In the hierarchical composite endpoint analysis, semaglutide achieved a higher number of wins relative to placebo (win ratio, 1.72; 95% CI, 1.37 to 2.15; P<0.001). The mean percentage change in CRP levels was a decrease of −43.5% with semaglutide versus −7.3% with placebo, corresponding to an estimated treatment ratio of 0.61 (95% CI, 0.51 to 0.72; *p* < 0.001). Subgroup analyses demonstrated consistent benefits across age, sex, and baseline NYHA classes [46].

The STEP-HFpEF DM trial focused on a similar HFpEF population but specifically enrolled 616 patients with coexisting T2DM. Over 52 weeks, participants received once-weekly subcutaneous semaglutide 2.4 mg or placebo. The dual primary endpoint and the hierarchical composite endpoint were the same as in the STEP-HFpEF trial. The average improvement in the KCCQ-CSS was 13.7 points with semaglutide compared to 6.4 points with placebo, resulting in an estimated difference of 7.3 points (95% CI, 4.1 to 10.4; *p* < 0.001). The mean percentage reduction in body weight was −9.8% with semaglutide versus −3.4% with placebo, corresponding to an estimated difference of −6.4% (95% CI, −7.6% to −5.2%; *p* < 0.001). Confirmatory secondary endpoints also favored semaglutide over placebo, including an estimated between-group difference in the change in 6 min walk distance of 14.3 m (95% CI, 3.7 to 24.9; *p* = 0.008), a win ratio for the hierarchical composite endpoint of 1.58 (95% CI, 1.29 to 1.94; *p* < 0.001), and an estimated treatment ratio for the change in CRP levels of 0.67 (95% CI, 0.55 to 0.80; *p* < 0.001) [47].

A subsequent pooled analysis integrated patient-level data from the STEP-HFpEF and STEP-HFpEF DM trials, collectively encompassing 1145 HFpEF patients. The dual primary endpoint was the change in the KCCQ Clinical Summary Score from baseline, a measure of HF-related quality of life, and the change in body weight. The improvements in the KCCQ-CSS and reductions in body weight from baseline to week 52 were significantly greater in the semaglutide group compared to the placebo group. The mean between-group difference in the KCCQ-CSS change from baseline to week 52 was 7.5 points (95% CI, 5.3 to 9.8; *p* < 0.0001), and the mean difference in body weight at week 52 was −8.4% (95% CI, −9.2 to −7.5; *p* < 0.0001). Confirmatory secondary endpoints also showed significant benefits with semaglutide, including a mean between-group difference in 6 min walk distance of 17.1 m (95% CI, 9.2 to 25.0), a win ratio for the hierarchical composite endpoint of 1.65 (95% CI, 1.42 to 1.91), and a treatment ratio for CRP reduction of 0.64 (95% CI, 0.56 to 0.72; *p* < 0.0001 for all comparisons). For the dual primary endpoints, the efficacy of semaglutide was consistent across various subgroups, including those defined by age, race, sex, BMI, SBP, baseline CRP levels, and LVEF. Taken together, this pooled analysis validated semaglutide’s position as a multifaceted therapy in HFpEF, offering improvements in symptoms, functional status, metabolic parameters, and hard clinical outcomes irrespective of diabetes status [48].

A prespecified analysis of the SELECT trial examined outcomes in patients with HF, with 4286 of 17,604 participants (24.3%) reporting a history of HF at baseline. Among these, 53% had HFpEF, 31.4% had HFrEF, and 15.5% had unclassified HF. The primary endpoints included time to first MACE, defined as CV death, nonfatal myocardial infarction, or nonfatal stroke; a composite HF outcome consisting of CV death, HF hospitalization, or urgent HF visits; CV death; and all-cause mortality. Secondary endpoints included the individual components of the primary outcomes and safety assessments. Semaglutide significantly improved all primary endpoints in participants with a history of HF compared to those without. It reduced the risk of MACE by 28% (HR 0.72; 95% CI: 0.60–0.87), the composite HF endpoint by 21% (HR 0.79; 95% CI: 0.64–0.98), CV death by 24% (HR 0.76; 95% CI: 0.59–0.97), and all-cause death by 19% (HR 0.81; 95% CI: 0.66–1.00). These benefits were observed in both the HFpEF and HFrEF groups, despite higher absolute event rates in the HFrEF subgroup. In patients with HFpEF, semaglutide reduced the risk of MACE (HR 0.69; 95% CI: 0.51–0.91) and the composite HF outcome (HR 0.75; 95% CI: 0.52–1.07), although reductions in CV death (HR 0.76; 95% CI: 0.55–1.05) and all-cause mortality (HR 0.78; 95% CI: 0.59–1.03) were not statistically significant. Similarly, in HFrEF patients, the HRs were 0.65 (95% CI: 0.49–0.87) for MACE, 0.79 (95% CI: 0.58–1.08) for the composite HF outcome, 0.77 (95% CI: 0.55–1.08) for CV death, and 0.82 (95% CI: 0.62–1.09) for all-cause mortality. The observed benefits were consistent across subgroups defined by baseline characteristics, including age, sex, BMI, NYHA class, and diuretic use. Additionally, semaglutide demonstrated a favorable safety profile, with fewer serious adverse events reported regardless of HF subtype. These findings reinforced semaglutide’s broad cardioprotective potential, extending beyond MACE reduction to encompass meaningful improvements in HF-related outcomes [49].

A recent post hoc pooled, participant-level analysis combined data from four placebo-controlled RCTs—SELECT, FLOW, STEP-HFpEF, and STEP-HFpEF DM trials—to evaluate the impact of once-weekly semaglutide on HF outcomes in patients with HFpEF/HFmrEF. Across these four studies, which enrolled diverse populations (patients with obesity-related HFpEF, those with ASCVD and overweight or obesity, and individuals with T2DM and CKD), investigators identified 3743 participants (16.8% of the combined 22,282 trial population) with HFpEF. Of these, 1914 were assigned to semaglutide and 1829 to placebo. Semaglutide doses varied across trials: 2.4 mg weekly in SELECT, STEP-HFpEF, and STEP-HFpEF DM, and 1.0 mg weekly in FLOW. The pooled results showed that semaglutide significantly reduced the risk of the composite endpoint of CV death or worsening HF events by approximately 31% (HR: 0.69; 95% CI: 0.53–0.89; *p* = 0.0045) relative to placebo, lowering event rates from 7.5% to 5.4% (ARR of ~2.1%, NNT of ~48). This reduction was driven mainly by a 41% lower risk of worsening HF events (hospitalizations or urgent visits) (HR: 0.59; 95% CI: 0.41–0.82; *p* = 0.0019). While there was no statistically significant effect on CV death alone (HR: 0.82; 95% CI: 0.57–1.16; *p* = 0.25), the meaningful decrease in worsening HF events marks an important finding for HFpEF management. Safety data supported semaglutide’s favorable profile, as fewer serious adverse events occurred in the semaglutide group (29.9%) compared to placebo (38.7%). These comprehensive, pooled findings highlight semaglutide’s potential of joining the GDMT of HFpEF [50].

#### 6.3.4. GIP/GLP-1RAs in Chronic HF

The SUMMIT trial involved 731 patients with HFpEF (LVEF ≥50%) and obesity (BMI ≥30 kg/m^2^) with or without T2DM to assess tirzepatide’s effects over a period of at least 52 weeks. The primary endpoint was a composite of CV death or worsening HF (hospitalization or urgent visit). Baseline medications included diuretics (74%), mineralocorticoid receptor antagonists (35%), and SGLT2is (17%). Over a median of 104 weeks, in the tirzepatide group, 36 patients (9.9%) experienced CV death or a worsening HF event, compared to 56 patients (15.3%) in the placebo group, leading to an HR of 0.62 (95% CI, 0.41 to 0.95; *p* = 0.026). This translated to an ARR of 5.4% and an NNT of 19. Worsening HF events were observed in 29 patients (8.0%) receiving tirzepatide and 52 patients (14.2%) on placebo (HR = 0.54; 95% CI, 0.34 to 0.85). After 52 weeks, the mean change in the KCCQ-CSS was 19.5±1.2 for the tirzepatide group, compared to 12.7±1.3 in the placebo group, resulting in a between-group difference of 6.9 points (95% CI, 3.3 to 10.6; *p* < 0.001). SUMMIT thus suggested that tirzepatide’s profound metabolic and weight benefits translate into measurable improvements in HFpEF outcomes irrespective of diabetes status [51].

### 6.4. Key Considerations for the Use of SGLT2is, GLP-1RAs, and GIP/GLP-1RAs in CVD

The extensive body of evidence from trials investigating SGLT2is and GLP-1RAs in CVD highlights their transformative impact on managing ASCVD, HF, and overall CV outcomes. SGLT2is significantly reduce the risk of HF hospitalization and CV death, establishing them as a GDMT pillar for HF management, independent of diabetes status. Trials such as DAPA-HF and EMPEROR-Reduced demonstrated substantial reductions in HF-related outcomes in patients with HFrEF, while the DELIVER and EMPEROR-Preserved trials extended these benefits to HFpEF. These findings underscore the versatility of SGLT2is in addressing the broad spectrum of HF phenotypes, with consistent benefits across key subgroups, including patients with varying renal function and without diabetes.

When using SGLT2is in treating HF, physicians should recognize the time-varying effects these agents have on critical clinical outcomes, which reflect both their immediate and longer-term impacts [52,53]. According to a recently published meta-analysis, the reduction in hospitalization for HF is substantial early in therapy, with a risk reduction of 42% (RR: 0.58, 95% CI: 0.48–0.71) at three months, which gradually decreases to 39% (RR: 0.61, 95% CI: 0.56–0.66) at six months, 33% (RR: 0.67, 95% CI: 0.65–0.70) at 12 months, 28% (RR: 0.72, 95% CI: 0.69–0.74) at 18 months, and 25% (RR: 0.75, 95% CI: 0.71–0.79) at 24 months. CV mortality, however, shows a more constant trend in reduction across time, with a 14% risk reduction at three months (RR: 0.86, 95% CI: 0.79–0.95) which plateaus to a robust 11% risk reduction (RR: 0.89, 95% CI: 0.82–0.96) at 18 and 24 months. All-cause mortality initially declines significantly, with a risk reduction of 11% (RR: 0.89, 95% CI: 0.81–0.98) at three and six months, but this effect diminishes beyond this period and does not maintain statistical significance [53].

These outcomes underscore the importance of recognizing the time-sensitive nature of SGLT2i effects in HF management. The substantial early benefits in reducing hospitalization and CV mortality highlight the urgency of initiating treatment promptly, as the relative risk reductions are the most pronounced in the first few months. However, the gradual decline in efficacy over time, particularly for HF hospitalization and all-cause mortality, suggests that their impact may plateau with prolonged use. This diminishing effect does not negate their long-term value but indicates the need to integrate SGLT2is into a broader, multifaceted treatment plan. Such an approach should address the evolving needs of patients over time, ensuring that other therapies complement the sustained, albeit attenuated, benefits of SGLT2 inhibitors. Understanding this dynamic allows clinicians to optimize treatment strategies, balancing immediate benefits with the necessity for ongoing interventions to improve long-term outcomes in HF [52,53].

For acute HF, the EMPULSE and SOLOIST-WHF trials provided critical insights. EMPULSE demonstrated significant improvements in decongestion outcomes, such as reductions in body weight and NT-proBNP, and enhanced overall clinical status in patients hospitalized for acute HF. SOLOIST-WHF, despite early termination due to funding issues, highlighted the ability of sotagliflozin to reduce the composite endpoint of CV death, HF hospitalizations, and urgent visits in high-risk patients with T2DM recently hospitalized for worsening HF. Together, these studies emphasize the potential role of SGLT2is in acute HF management, suggesting that their benefits extend to this high-risk and underserved patient population.

Based on the landmark meta-analyses demonstrating the significant CV benefits of SGLT2is and GLP-1RAs in patients with T2DM, the 2023 European Society of Cardiology guidelines for the management of T2DM in patients with CVD issued a class I recommendation for the use of these agents in patients with T2DM and established ASCVD, regardless of other anti-diabetic medications or glycemic targets [32,33,49]. Furthermore, a class IIb recommendation was provided for the use of SGLT2is and GLP-1RAs in patients with diabetes at high CV risk (SCORE2-Diabetes >10% in the absence of ASCVD or severe target organ damage) [54].

The SELECT trial was transformative for the management of ASCVD. This landmark study demonstrated a 20% reduction in MACE with semaglutide in overweight or obese patients with established ASCVD without diabetes. Its results prompted the 2024 European Society of Cardiology guidelines for chronic coronary syndromes to issue a class IIa recommendation for semaglutide in overweight or obese patients with established ASCVD without diabetes, recognizing its ability to improve hard CV endpoints [55].

In HF, the STEP HFpEF program, including STEP-HFpEF and STEP-HFpEF DM, provided groundbreaking data on GLP-1RAs, particularly semaglutide, in HFpEF populations. These trials demonstrated significant improvements in weight loss, quality of life, exercise capacity, and prognosis. The prespecified HF analysis of the SELECT trial further highlighted semaglutide’s potential by demonstrating significant reductions in hard clinical endpoints for both HFpEF and HFrEF. A pooled meta-analysis integrating data from the STEP HFpEF, STEP HFpEF DM, SELECT, and FLOW trials confirmed that semaglutide can reduce the composite endpoint of CV death or worsening HF in HFpEF. These findings underscore the potential of semaglutide to address HF-related morbidity, particularly in HFpEF.

The SUMMIT trial showed promising results for tirzepatide in patients with HFpEF and obesity, irrespective of T2DM status, achieving a significant reduction in the composite endpoint of CV death or HF hospitalization. It is interesting that it reached statistical significance despite its small sample size. These findings suggest the potential for GIP/GLP-1RAs like tirzepatide to emerge as distinct GDMT options for HFpEF with overweight or obesity. Future guidelines may consider incorporating these agents based on their growing evidence base.

Despite these advancements, important gaps remain. Specific trials investigating the efficacy of GLP-1RAs in phase III event-driven RCTs with strict inclusion criteria based on LVEF (e.g., <40% and >40%) are lacking. Furthermore, a study like the SUMMIT is necessary in patients with LVEF <40% to investigate the effects of tirzepatide on patients with obesity and HFrEF. Additionally, the potential benefits of GLP-1RAs and dual GIP/GLP-1RAs in patients with HF or ASCVD but without overweight/obesity remain unexplored and represent an important avenue for future research. Evaluating these agents across the spectrum of HF phenotypes, irrespective of obesity status, will be crucial to defining their full therapeutic potential in cardiology. Finally, an important limitation of the SUMMIT trial was that only 17% of patients were receiving SGLT2 inhibitors at enrollment. Consequently, the potential additive effects of GIP/GLP-1 receptor agonists in combination with SGLT2 inhibitors in HFpEF remain unexplored and warrant further investigation.

## 7. CKD

Similarly to their cardioprotective effects, the first insights into the renoprotective effects of GLP-1RAs and SGLT2is emerged from trials conducted on patients with diabetes [36,37]. Specifically, a large meta-analysis on SGLT2 inhibitors found that these medications significantly reduced the risk of kidney outcomes (HR: 0.62; 95% CI: 0.56–0.70; *p* = 0.09) in patients with T2DM. The association with kidney outcomes was consistent regardless of the presence or absence of ASCVD (*p* interaction = 0.73) [36]. Regarding GLP-1RAs, a large meta-analysis on patients with T2DM demonstrated that these medications reduced the composite kidney outcome by 21% (HR: 0.79; 95% CI 0.73–0.87, *p* < 0.0001) [37]. These meta-analyses of RCTs provided the rationale for conducting further studies to evaluate the effects of SGLT2is in CKD irrespective of diabetes and GLP-1RAs in diabetic CKD, as discussed below.

### 7.1. SGLT2is

The DAPA-CKD trial enrolled 4304 patients with CKD (eGFR 25–75 mL/min/1.73 m^2^, median ~43 mL/min/1.73 m^2^) and a urinary albumin-to-creatinine ratio (UACR) of 200 to 5000 mg/g, of whom approximately 67.5% had T2DM. All were on standard therapy, including RASIs in >97%. Over a median of 2.4 years, dapagliflozin 10 mg daily lowered the primary composite (≥50% eGFR decline; end-stage kidney disease—ESKD; or renal/CV death) by 39% (HR: 0.61; 95% CI: 0.51–0.72; *p* < 0.001), reducing event rates from 14.5% to 9.2% (ARR ~5.3%, NNT ~19). Secondary endpoints showed a significant reduction in CV death or HF hospitalization (HR: 0.71; 95% CI: 0.55–0.92; *p* = 0.009) and a 31% drop in all-cause mortality (HR: 0.69; 95% CI: 0.53–0.88; *p* = 0.004). Subgroup analyses indicated robust benefits across age groups, sex, geographic location, and diabetic and non-diabetic status (T2DM: HR = 0.64; 95% CI: 0.52–0.79, non-diabetic: HR = 0.50, 95% CI: 0.35–0.72), as well as various levels of baseline kidney function, albuminuria, and blood pressure. These findings established dapagliflozin as a cornerstone therapy in CKD irrespective of T2DM status, representing a major shift toward SGLT2is for both renal and CV risk reduction [56].

The EMPA-KIDNEY trial enrolled 6609 patients with CKD—defined by an eGFR between 20 and 45 mL/min/1.73 m^2^ or 45 and <90 mL/min/1.73 m^2^ with a UACR of 200 to 5000 mg/g—with (46.2%) and without (53.8%) diabetes mellitus, of whom approximately 85% were on RASIs. The median follow-up was about two years. The primary endpoint was the composite of kidney disease progression (defined as the occurrence of any of the following: ESKD, a sustained reduction in eGFR to less than 10 mL/min/1.73 m^2^, a sustained decline in eGFR of at least 40% from baseline, or death due to renal causes) or CV death. Empagliflozin 10 mg once daily reduced this primary endpoint by 28% compared to placebo (HR: 0.72; 95% CI: 0.64–0.82; *p* < 0.001), with event rates of approximately 13.1% vs. 16.9%—an ARR of 3.8% and an NNT of 26. Subgroup analyses indicated robust benefits across diabetic and non-diabetic status (diabetes mellitus: HR = 0.64; 95% CI: 0.54–0.77, non-diabetic: HR = 0.82, 95% CI: 0.68–0.99), as well as various levels of baseline kidney function and albuminuria. Together with DAPA-CKD, EMPA-KIDNEY demonstrated the class effect of SGLT2is in the management of CKD irrespective of diabetes status [57].

### 7.2. GLP-1RAs

The FLOW trial enrolled 3533 patients with T2DM and CKD, defined by a sustained eGFR decline and albuminuria on standard care. Participants were randomized to once-weekly subcutaneous semaglutide 1 mg or placebo and followed up with for a median of 3.5 years. The semaglutide group experienced a 24% reduction in the risk of a primary outcome event compared to the placebo group, with 331 (18.7%) versus 410 (23.2%) first events (HR, 0.76; 95% CI, 0.66 to 0.88; *p* = 0.0003). This translated to an ARR of 4.5% and an NNT of 22.2. Similar findings were observed for the composite kidney-specific components of the primary outcome (HR, 0.79; 95% CI, 0.66 to 0.94) and for CV death (HR, 0.71; 95% CI, 0.56 to 0.89). All confirmatory secondary outcomes also showed favorable results for semaglutide. The mean annual eGFR slope was 1.16 mL/min/1.73 m^2^ less steep in the semaglutide group, indicating a slower rate of decline (*p* < 0.001). Additionally, the risk of MACE was reduced by 18% (HR, 0.82; 95% CI, 0.68 to 0.98; *p* = 0.029), and the risk of death from any cause was 20% lower (HR, 0.80; 95% CI, 0.67 to 0.95; *p* = 0.01). Taken together, FLOW positioned semaglutide as a potent pillar for the management of diabetic nephropathy and its associated CV risk [58].

A prespecified analysis of HF outcomes from the FLOW trial provided additional insights into the therapeutic potential of semaglutide in HF in patients with diabetic CKD. HF was present at baseline in 342 participants (19.4%) in the semaglutide group and 336 participants (19.0%) in the placebo group. The primary endpoint was the composite of HF events (new onset or worsening of HF leading to an urgent visit or hospital admission, with initiation of or intensified diuretic/vasoactive therapy) or CV death. Across the entire trial population, semaglutide significantly improved time to first HF events or CV death (HR: 0.73; 95% CI: 0.62–0.87; *p* = 0.0005), time to first HF events alone (HR: 0.73; 95% CI: 0.58–0.92; *p* = 0.0068), and time to CV death alone (HR: 0.71; 95% CI: 0.56–0.89; *p* = 0.0036). The reduction in risk for the composite HF outcome was comparable between those with HF at baseline (HR: 0.73; 95% CI: 0.54–0.98; *p* = 0.0338) and those without (HR: 0.72; 95% CI: 0.58–0.89; *p* = 0.0028) [59].

### 7.3. Key Considerations for the Use of SGLT2is and GLP-1RAs in CKD

The trials investigating SGLT2is and GLP-1RAs in CKD have significantly reshaped our understanding of renal protection and cardiometabolic risk management. SGLT2is have emerged as foundational therapies for CKD, irrespective of diabetes status, while GLP-1RAs have shown promise in diabetic CKD populations.

The DAPA-CKD and EMPA-KIDNEY trials provided robust evidence supporting the role of SGLT2is in CKD, offering both renal and CV protection independent of diabetes status. The FLOW trial gave critical insights into the efficacy of GLP-1RAs in diabetic CKD, specifically in diabetic populations.

A recently published systematic review and meta-analysis of 13 RCTs (29,614 participants) demonstrated that SGLT2is significantly reduced the risks of all-cause death (RR 0.85, 95% CI 0.74 to 0.98), CV death (RR 0.84, 95% CI 0.74 to 0.96), kidney failure (RR 0.68, 95% CI 0.60 to 0.77), nonfatal myocardial infarction (RR 0.75, 95% CI 0.60 to 0.93), nonfatal stroke (RR 0.73, 95% CI 0.57 to 0.94), and hospital admissions for HF (RR 0.68, 95% CI 0.60 to 0.78) in adults with CKD, regardless of diabetes or HF status. Absolute benefits were stratified by baseline risk: high-risk groups experienced reductions of 48 all-cause deaths, 58 kidney failures, and 25 hospitalizations for HF per 1000 individuals over five years. Low-risk groups experienced reductions of seven all-cause deaths and four hospitalizations for HF but no effect on kidney failure per 1000 individuals over five years. Harms included increased risks of genital infections (RR 2.66, 95% CI 2.07 to 3.42), ketoacidosis (RR 2.27, 95% CI 1.30 to 3.95), and symptomatic hypovolemia (RR 1.29, 95% CI 1.15 to 1.44), though absolute rates were small [60].

These findings formed the basis of the BMJ clinical practice guidelines on SGLT2is for adults with CKD. The guideline panel, using evidence from this review, provided stratified recommendations according to risk categories defined by the Kidney Disease Improving Global Outcomes (KDIGO) system. For adults at low or moderate risk of CKD progression and complications, the panel suggested SGLT2i use (weak recommendation in favor). For adults at high or very high risk, the panel strongly recommended SGLT2i therapy. These recommendations are applicable irrespective of type 2 diabetes status and emphasize the balance of benefits and harms across risk strata. These guidelines highlight the need for clinicians to appropriately risk-stratify patients using eGFR and albuminuria levels, alongside shared decision-making, to tailor therapy to individual patient profiles [61].

Notably, while the absolute benefits of SGLT2i therapy are the most pronounced in high-risk populations, initiating treatment earlier in the course of CKD significantly extends the time to kidney failure and the need for kidney replacement therapy. For example, an analysis of the EMPA-KIDNEY trial indicated that starting therapy at an eGFR of 85 mL/min/1.73 m^2^ can delay the onset of kidney failure by up to 26.6 years, compared to only 1.9 years if therapy begins at an eGFR of 20 mL/min/1.73 m^2^. This delay translates to avoiding over 300 hemodialysis sessions per patient when treatment starts at an eGFR of 20 mL/min/1.73 m^2^ and over 4000 sessions per patient when initiated at an eGFR of 85 mL/min/1.73 m^2^. Beyond reducing the frequency of dialysis, early intervention mitigates the associated patient and healthcare burden and environmental impact, including transportation costs, energy and water consumption, and plastic waste generation from dialysis supplies. Even for individuals who may not progress to ESKD, preserving the eGFR through early SGLT2i use enhances CV health, reduces mortality risks, and improves the quality of life. These findings underscore the critical importance of early risk stratification and the timely initiation of therapy to maximize both clinical and systemic benefits across CKD populations [62].

According to the KDIGO 2024 guidelines, it is strongly recommended to treat patients with T2DM, CKD, and an eGFR ≥20 mL/min per 1.73 m^2^ with an SGLT2 inhibitor (1A). Similarly, in adults with non-diabetic CKD, SGLT2 inhibitors are recommended for those with an eGFR ≥20 mL/min per 1.73 m^2^ and UACR >200 mg/g (≥20 mg/mmol) or for those with HF, regardless of albuminuria levels (1A). For adults with an eGFR of 20–45 mL/min per 1.73 m^2^ and a UACR <200 mg/g (<20 mg/mmol), SGLT2 inhibitors are suggested as a treatment option (2B). Additionally, the KDIGO guidelines suggest the use of a nonsteroidal mineralocorticoid receptor antagonist (finerenone) for adults with T2DM, an eGFR >25 mL/min per 1.73 m^2^, normal serum potassium levels, and UACR >30 mg/g despite the maximum tolerated dose of RASIs (2A) [63].

The incorporation of findings from trials like DAPA-CKD, EMPA-KIDNEY, FIDELIO-DKD, and FIGARO-DKD into clinical guidelines has already begun to transform practice paradigms. In light of the robust evidence from the FLOW trial, future guidelines should consider expanding recommendations for GLP-1RAs to position them as an emerging fourth renoprotective pillar alongside RASIs, SGLT2is, and finerenone, particularly in the management of diabetic CKD.

An important consideration when a new pillar emerges is the added benefit to existing treatments. Evidence from a combined analysis of the CREDENCE (*n* = 4401) and FIDELIO-DKD (*n* = 5734) trials underscores the significant cumulative effect of finerenone when used alongside SGLT2is. This combination demonstrated a 50% reduction in the risk of a composite endpoint (doubling of serum creatinine, progression to ESKD, or mortality due to kidney failure) (HR 0.50; 95% CI: 0.44, 0.57). For a 50-year-old individual, this translated to an event-free survival of 16.7 years (95% CI: 18.1, 21.0) with combination therapy, compared to 10.0 years (95% CI: 6.8, 12.3) with standard treatment using angiotensin-converting enzyme inhibitors or angiotensin receptor blockers, resulting in a gain of 6.7 years (95% CI: 5.5, 7.9) [64].

Regarding the potential additive benefit of GLP-1RAs to SGLT2is, a sub-analysis of the FLOW trial evaluated semaglutide in participants with T2DM and CKD stratified by baseline SGLT2i use. The primary outcome—kidney failure, ≥50% eGFR decline, kidney death, or CV death—occurred in 41/277 (14.8%) participants on semaglutide versus 38/273 (13.9%) on placebo among those using SGLT2is at baseline (HR 1.07; 95% CI: 0.69–1.67; *p* = 0.755) and in 290/1490 (19.5%) versus 372/1493 (24.9%) participants not on SGLT2is (HR 0.73; 95% CI: 0.63–0.85; *p* < 0.001). Although semaglutide significantly reduced the risk of the primary outcome in participants not on SGLT2is, the *p* interaction (0.109) was not significant. This indicates that semaglutide has potential independent renal benefits, though the trial was underpowered to detect these additive effects on SGLT2is. The above should be confirmed in future well-powered studies. Meanwhile, semaglutide’s benefits to MACE and all-cause mortality were consistently significant across SGLT2i use, with *p* interaction = 0.741 and 0.901, respectively. These findings underscore semaglutide’s additive CV benefits to baseline SGLT2i use [65].

Another important recently published finding is the added benefit of triple therapy with SGLT2is, finerenone, and GLP-1RAs in patients with T2DM and a UACR ≥30 mg/g compared to RASI use alone. Triple therapy reduced the risk of MACE, including nonfatal myocardial infarction, nonfatal stroke, and CV death (HR 0.65; 95% CI: 0.55–0.76), yielding an absolute risk reduction of 4.4% (95% CI: 3.0–5.7) over three years and an NNT of 23. For a 50-year-old patient initiating triple therapy, projected MACE-free survival was 21.1 years compared to 17.9 years for conventional care, an increase of 3.2 years (95% CI: 2.1–4.3). Additional gains included extended survival free from HF hospitalization (3.2 years; 95% CI: 2.4–4.0), CKD progression (5.5 years; 95% CI: 4.0–6.7), CV death (2.2 years; 95% CI: 1.2–3.0), and all-cause death (2.4 years; 95% CI: 1.4–3.4). These findings suggest that this triple combination may provide significant clinical benefits for patients with T2DM and even mild albuminuria, further underscoring the importance of utilizing all four pillars of CKD therapy [66].

Despite these advancements, key gaps remain. SGLT2is have established broad applicability across diabetic and non-diabetic CKD populations, yet the evidence base for GLP-1RAs in non-diabetic CKD is yet absent. Dedicated trials assessing the renoprotective effects of GLP-1RAs and dual GIP/GLP-1RAs in non-diabetic CKD populations are needed to delineate their full therapeutic potential. Furthermore, any potential additive benefits of GLP-1RAs to the combination of RASI, SGLT2is, and finerenone should also be investigated.

Finally, there is a significant gap in evidence regarding the use of SGLT2is, GLP-1RAs, and dual GIP/GLP-1RAs for patients with an eGFR below 20 mL/min/1.73 m^2^ and in ESKD. According to the KDIGO 2024 guidelines, SGLT2is should not be initiated in patients with an eGFR below 20 mL/min/1.73 m^2^. However, once treatment with an SGLT2i is initiated, the guidelines state that it is generally reasonable to continue therapy even if the eGFR falls below 20 mL/min/1.73 m^2^, provided the treatment is well tolerated and kidney replacement therapy has not been initiated [61].

Notably, in patients with ESKD undergoing dialysis, where conventional therapies often fail to mitigate the substantial CV risk, SGLT2is and GLP-1RAs may offer both direct and indirect benefits [66,67].

Regarding SGLT2is in ESKD, mechanistic studies indicate that these agents can lower intracellular sodium and calcium levels in cardiomyocytes, reduce oxidative and endoplasmic reticulum stress, suppress inflammation, and regulate autophagy, potentially improving cardiac function. Additionally, indirect CV benefits may arise from their capacity to preserve residual kidney function, mitigate anemia by increasing erythropoietin production, and reduce inflammatory pathways. These effects could collectively decrease the burden of HF, myocardial remodeling, and vascular dysfunction, which are prevalent and critical challenges in the ESKD population. Despite their diminished glycosuric and natriuretic effects in advanced CKD and ESKD, SGLT2 inhibitors are being actively studied for their non-glucose-mediated benefits. Several ongoing trials are evaluating outcomes such as heart failure hospitalization, all-cause mortality, and cardiac imaging biomarkers in this population. These studies aim to establish the safety and efficacy of SGLT2 inhibitors, potentially redefining therapeutic strategies for ESKD patients with high CV risk. The promising preclinical and emerging clinical evidence highlights the need for larger RCTs to determine their role as a standard therapy in dialysis-dependent ESKD [67].

Regarding GLP-1RAs in ESKD, a recently published meta-analysis evaluated their safety and efficacy in patients with T2DM and advanced CKD, including ESKD. The analysis included eight studies with a total of 27,639 patients and found no significant difference in one-year mortality between GLP-1RA and control groups. However, GLP-1RAs significantly reduced the cardiothoracic ratio (standardized mean difference [SMD]: −1.2%; 95% CI: −2.0 to −0.4) and serum pro-BNP levels (SMD: −335.9 pmol/L; 95% CI: −438.9 to −232.8). They also lowered mean blood glucose levels (SMD: −1.1 mg/dL; 95% CI: −1.8 to −0.3) and led to notable weight reductions (SMD: −2.2 kg; 95% CI: −2.9 to −1.5). While there was no significant reduction in SBP or HbA1c, GLP-1RAs were not associated with an increased risk of hypoglycemia. Gastrointestinal side effects were more frequent, with a 3.8-fold higher risk of nausea and a 35.7-fold higher risk of vomiting. Despite these side effects, the findings suggest that GLP-1RAs are generally safe and provide meaningful CV and metabolic benefits for this high-risk population [68].

## 8. MASLD

Beyond the heart and kidneys, SGLT2is, and particularly GLP-1RAs and GIP/GLP-1RAs, have shown promise in MASLD (formerly known as non-alcoholic fatty liver disease—NAFLD) and MASH (formerly known as non-alcoholic steatohepatitis—NASH). Metabolic dysregulation, insulin resistance, and chronic low-grade inflammation are central to the pathogenesis of MASLD and its progression to MASH and advanced fibrosis.

Regarding SGLT2is, a recently published meta-analysis showed that these drugs were superior to placebo in reducing liver fat content, as well as decreasing the levels of liver enzymes. Notably, network meta-analysis indicated that SGLT2is were more effective than GLP-1RAs in reducing transaminase levels. However, there is no robust evidence that SGLT2is can lead to a histological improvement in MASLD/MASH [69].

Regarding GLP-1RAs, a landmark, 72-week, phase 2 trial published in 2021 evaluated once-daily subcutaneous semaglutide 0.1, 0.2, and 0.4 mg daily in patients with biopsy-confirmed MASH and F2–F3 fibrosis. The primary endpoint was MASH resolution without the worsening of fibrosis. At the highest dose (0.4 mg daily), semaglutide achieved MASH resolution rates of approximately 59% compared to 17% with placebo (*p* < 0.001), representing a more than threefold improvement. While the trial did not reach its endpoint for significant fibrosis improvement (F2–F3 stages), it did show numerical trends in reducing fibrosis progression, warranting further investigation. In addition, participants experienced meaningful metabolic improvements, including weight reduction, improved glycemic control in patients with T2DM, and reduced liver enzyme levels (ALT, AST), as well as significant decreases in MRI-PDFF-measured liver steatosis [70].

Beyond the above trial, several other studies have explored the effects of various GLP-1RAs in managing MASLD and MASH. A recently published meta-analysis of RCTs demonstrated that GLP-1RAs were significantly more effective than placebo in resolving MASH (RR: 2.48; 95% CI: 1.86–3.30), reducing liver fat content, and lowering liver enzyme levels [69].

On 1 November 2024, Novo Nordisk announced the headline results from part 1 of the pivotal phase 3 ESSENCE trial, a 240-week, double-blinded study evaluating the efficacy of once-weekly subcutaneous semaglutide 2.4 mg in adults with MASH and moderate to advanced liver fibrosis (F2 or F3). The primary endpoints focused on liver histological improvement and the resolution of steatohepatitis without worsening fibrosis after 72 weeks. The trial successfully met its primary endpoints. At week 72, 37.0% of patients treated with semaglutide 2.4 mg achieved improvement in liver fibrosis with no worsening of steatohepatitis, compared to 22.5% on placebo. Additionally, 62.9% of participants achieved the resolution of steatohepatitis with no worsening of liver fibrosis, compared to 34.1% on placebo. These findings mark a significant step forward in addressing an unmet medical need for patients with MASH, a disease that often progresses silently to cirrhosis or liver cancer if left untreated. In terms of safety, semaglutide 2.4 mg demonstrated a well-tolerated profile consistent with previous clinical trials. Martin Holst Lange, Executive Vice President and Head of Development at Novo Nordisk, emphasized the following potential impact: “Among people with overweight or obesity, one in three live with MASH. This has a serious impact on their health and represents a significant unmet need. Novo Nordisk anticipates regulatory filings in the U.S. and EU by the first half of 2025, with detailed results from ESSENCE scheduled for presentation at an upcoming scientific conference in 2024. The second part of the ESSENCE trial, aimed at evaluating long-term outcomes and liver-related events over 240 weeks, will continue through 2029 [71,72]”.

The SYNERGY-NASH trial recruited 190 patients with biopsy-confirmed MASH and F2–F3 fibrosis, evaluating once-weekly subcutaneous tirzepatide (5, 10, or 15 mg weekly) versus placebo over 52 weeks. The primary endpoints included MASH resolution without worsening fibrosis and ≥1-stage fibrosis improvement. Tirzepatide led to MASH resolution in 44% (5 mg), 56% (10 mg), and 62% (15 mg) vs. ~10% with placebo (*p* < 0.001) and ≥1-stage fibrosis improvement in ~50% of treated patients compared to ~30% of placebo recipients (*p* < 0.001). Subgroup analyses revealed consistent hepatic benefits irrespective of baseline fibrosis severity and diabetic status. SYNERGY-NASH positioned tirzepatide as a promising multifunctional agent, potentially altering the natural history of MASH through significant histological and metabolic improvements [73].

As a result, GLP-1RAs and GIP/GLP-1RAs have shown remarkable potential in managing MASLD and MASH, addressing both hepatic and metabolic components. Semaglutide has demonstrated substantial efficacy in resolving steatohepatitis and improving liver fibrosis, with associated benefits in weight reduction, glycemic control, and liver enzyme levels. Similarly, tirzepatide has shown significant promise in resolving steatohepatitis and improving fibrosis, highlighting its dual impact on liver health and metabolic outcomes.

Despite these promising developments, several challenges and gaps remain. The long-term impact of these therapies on clinical outcomes such as cirrhosis progression, hepatocellular carcinoma incidence, and liver-related mortality remains unclear and warrants further investigation. Trials like ESSENCE Part 2, which focus on long-term outcomes over several years, will be crucial in defining the role of GLP-1RAs and GIP/GLP-1RAs in the MASLD spectrum. Additionally, the cost and accessibility of these therapies, particularly in resource-limited settings, pose barriers to widespread adoption.

Looking ahead, integrating these agents into clinical practice will require a paradigm shift in managing MASLD and MASH, with a focus on early detection and comprehensive metabolic management. The 2024 EASL–EASD–EASO Clinical Practice Guidelines on the management of MASLD recommend liver-directed thyroid hormone receptor agonists (resmetirom) for patients with non-cirrhotic MASH and liver fibrosis stage 2 or higher. However, these guidelines state that “in the absence of a formal demonstration of histological improvement in large, well conducted, phase III trials, glucagon-like peptide 1 receptor agonists (GLP1RA) cannot currently be recommended as MASH-targeted therapies”. In light of the ongoing ESSENCE trial, a future update of these guidelines should consider recommending the inclusion of GLP-1RAs and dual GIP/GLP-1RAs in the management of MASLD and MASH, alongside resmetirom [74].

## 9. A Unifying Paradigm: Comprehensive Cardiometabolic Care

Collectively, the above robust evidence positions SGLT2is, GLP-1RAs, and dual GIP/GLP-1RAs as treatment pillars with multifaceted effects on CRHM syndrome. These agents improve glycemic control, reduce body weight, reduce blood pressure, reduce AHI, improve hard endpoints in HF and ASCVD and CKD, and improve the course of MASLD/MASH (Table 1).

**Figure 4:**
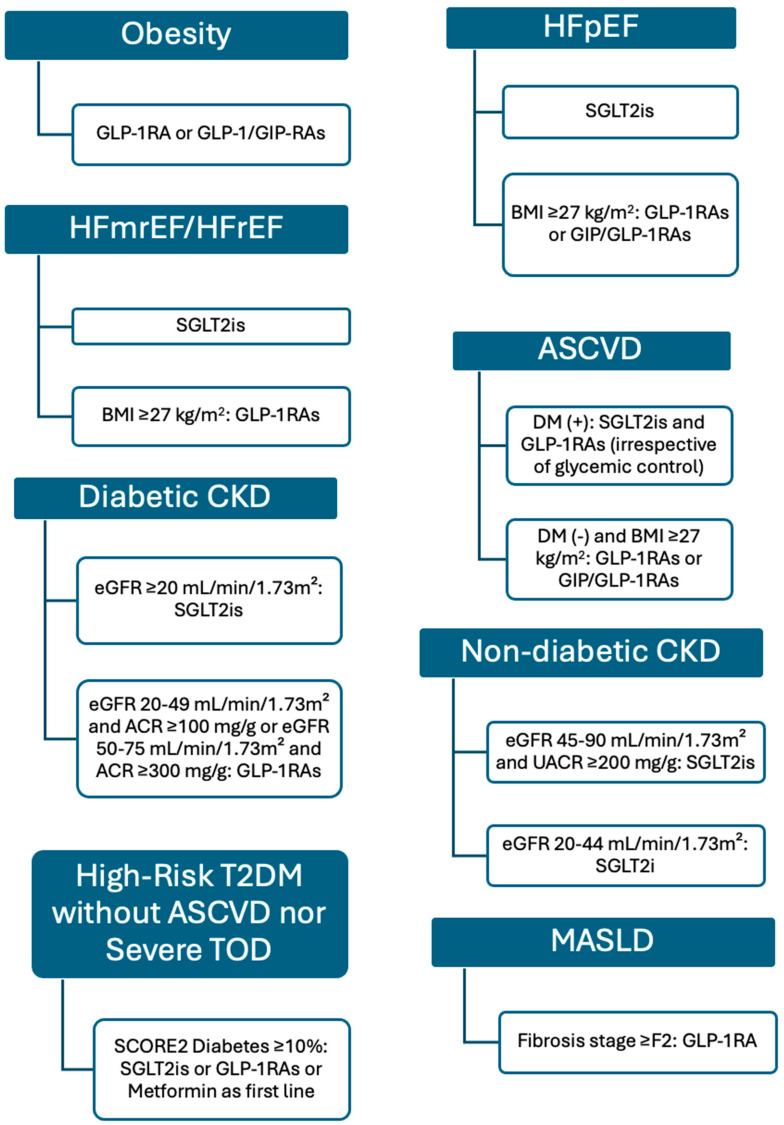
A proposed algorithm for the use of SGLT2is, GLP-1RAs, and GIP/GLP-1RAs for the management of CRHM syndrome. This illustration shows our proposed algorithm for the use of established SGLT2is, GLP-1RAs, and GIP/GLP-1RAs across CRHM syndrome based on the available guidelines and phase III randomized controlled trials. The use of GLP-1RAs and GIP/GLP-1RAs in HF, diabetic CKD and MASLD has not yet been recommended by guidelines because the evidence from trials is very recent. Abbreviations ASCVD (atherosclerotic cardiovascular disease); BMI (body mass index); CKD (chronic kidney disease); DM (Diabetes Mellitus); eGFR (estimated glomerular filtration rate); GIP (Glucose-Dependent Insulinotropic Polypeptide); GLP-1RA (glucagon-like peptide-1 receptor agonist); HF (heart failure); HFmrEF (heart failure with mildly reduced ejection fraction); HFpEF (heart failure with preserved ejection fraction); HFrEF (heart failure with reduced ejection fraction); MASLD (metabolic dysfunction-associated steatotic liver disease); SCORE2 (Systematic Coronary Risk Evaluation 2); SGLT2is (sodium–glucose cotransporter-2 inhibitor); TOD (target organ damage); T2DM (Type 2 Diabetes Mellitus); UACR (urinary albumin-to-creatinine ratio).

Our proposed algorithm for the management of patients with CRHM syndrome is illustrated in Figure 4.

The interconnected pathophysiology and shared medical management of CRHM syndrome underscore the need for a unified field—such as cardiometabolic medicine—to reduce healthcare fragmentation, improve patients’ outcomes, and promote research. By integrating these novel anti-diabetic therapies across all relevant indications early and comprehensively, cardiometabolic specialists can proactively prevent the progression of CRHM syndrome to advanced stages or reduce complications in those with established diseases. This approach can improve survival rates, reduce hospitalizations and healthcare costs, and enhance patients’ quality of life across the entire CRHM spectrum. Furthermore, the emerging roles of hormonal therapies in the management of HF, including testosterone, growth hormone, ghrelin, and triiodothyronine, give rise to the potential of incorporating endocrine therapies in cardiometabolic practice [75,76,77,78,79].

As further event-driven trials emerge and new indications are explored, it will be essential for physicians, researchers, and policymakers to collaborate in crafting updated guidelines and consensus statements, forming new training curricula, and promoting transdisciplinary healthcare models. This holistic, patient-centered approach can redefine the standard of care, transforming how we prevent, diagnose, and treat the global cardiometabolic pandemic.

## 10. Conclusions

The scientific evidence is unequivocal: SGLT2is, GLP-1RAs, and dual GIP/GLP-1RAs have radically redefined the boundaries of what is possible in the management of CRHM syndrome. Through meticulously designed, event-driven phase III RCTs, these therapies have demonstrated unparalleled efficacy in reducing MACE, HF hospitalizations, and CKD progression and enabling clinically meaningful weight loss and metabolic improvements. Integrating these agents into daily practice signifies a new era in which the boundaries separating the heart, kidney, liver, and metabolic pathways blur, enabling the healthcare community to confront the cardiometabolic pandemic head-on with high precision. As this shift accelerates, the overarching goal of improving longevity, organ function, and quality of life for billions of patients with non-communicable diseases becomes ever more attainable.

## Figures and Tables

**Table 1:** The proven benefits of SGLT2is, GLP-1RAs, and GIP/GLP-1RAs for the management of CRHM syndrome. This table summarizes the established and potential benefits of SGLT2is, GLP-1RAs, and GIP/GLP-1RAs across CRHM syndrome. ASCVD (atherosclerotic cardiovascular disease); CKD (chronic kidney disease); CRHM (Cardiovascular–Renal–Hepatic–Metabolic); HF (heart failure); HFpEF (heart failure with preserved ejection fraction); HFmrEF (heart failure with mildly reduced ejection fraction); MASLD (metabolic dysfunction-associated steatotic liver disease); OSA (obstructive sleep apnea); T2DM (type 2 diabetes mellitus).

Diseases	SGLT2is	GLP-1RAs	GIP/GLP-1RAs
T2DM	Multiple trials and meta-analyses		
Obesity	▪ T2DM: multiple trials (not primary endpoint) ▪ Non-diabetic patients: Lack of evidence	▪ STEP program ▪ SCALE program	▪ SURMOUNT program
Arterial Hypertension	▪ T2DM: multiple trials (not primary endpoint) ▪ Non-diabetic patients: Lack of evidence	▪ T2DM: multiple trials (not primary endpoint) ▪ Non-diabetic patients: Lack of evidence	▪ T2DM: multiple trials (not primary endpoint) ▪ Non-diabetic patients: Lack of evidence
HFpEF	▪ EMPEROR-Preserved ▪ DELIVER	▪ STEP-HFpEF program ▪ Prespecified analysis of SELECT ▪ Prespecified analysis of FLOW ▪ Pooled analysis of STEP-HFpEF, STEP-HFpEF DM, SELECT, and FLOW	▪ SUMMIT trial
HFrEF	▪ EMPEROR-Reduced ▪ DAPA-HF	▪ Prespecified analysis of SELECT ▪ Prespecified analysis of FLOW	▪ Lack of evidence
Acute HF	▪ SOLOIST-WHF ▪ EMPULSE	▪ Lack of evidence	▪ Lack of evidence
ASCVD	▪ T2DM: multiple trials and a meta-analysis ▪ Non-diabetic patients: Lack of evidence	▪ T2DM: multiple trials and a meta-analysis ▪ Non-diabetic patients: SELECT	▪ Lack of evidence
CKD	▪ T2DM: multiple trials and a meta-analysis ▪ Non-diabetic patients ○ EMPA-KIDNEY ○ DAPA-CKD	▪ T2DM: FLOW ▪ Non-diabetic patients: Lack of evidence	▪ Lack of evidence
MASLD	▪ No evidence of histologic improvement	▪ Multiple trials (notably ESSENCE) and a meta-analysis	▪ SYNEGY-NASH
OSA	▪ Lack of evidence	▪ Scoping review (low evidence)	▪ SURMOUNT-OSA

## Abbreviations

The following abbreviations are used in this manuscript:

AHI	Apnea–hypopnea index
ALT	Alanine Aminotransferase
AMPK	AMP-activated protein kinase
ARR	Absolute risk reduction
ASCVD	Atherosclerotic cardiovascular disease
AST	Aspartate Aminotransferase
BMI	Body mass index
CAD	Coronary artery disease
CI	Confidence interval
CKD	Chronic kidney disease
CM	Cardiometabolic
CKM	Cardiovascular–Kidney–Metabolic
CRHM	Cardiovascular–Renal–Hepatic–Metabolic
CRP	C-Reactive Protein
CVD	Cardiovascular disease
DBP	Diastolic blood pressure
EF	Ejection fraction
eGFR	Estimated glomerular filtration rate
ESKD	End-stage kidney disease
GDMT	Guideline-directed medical therapy
GIP	Glucose-Dependent Insulinotropic Polypeptide
GLP-1RA	Glucagon-like peptide-1 receptor agonist
HbA1c	Glycated hemoglobin
HF	Heart failure
HFmrEF	Heart failure with mildly reduced ejection fraction
HFpEF	Heart failure with preserved ejection fraction
HR	Hazard ratio
KCCQ-CSS	Kansas City Cardiomyopathy Questionnaire Clinical Summary Score
LVEF	Left ventricular ejection fraction
MACE	Major adverse cardiovascular events
MASLD	Metabolic dysfunction-associated steatotic liver disease
MASH	Metabolic dysfunction-associated steatohepatitis
MetS	Metabolic syndrome
MRI-PDFF	Magnetic Resonance Imaging–Proton Density Fat Fraction
NASH	Non-alcoholic steatohepatitis
NAFLD	Non-alcoholic fatty liver disease
NNT	Number needed to treat
NO	Nitric oxide
NYHA	New York Heart Association
OSA	Obstructive sleep apnea
PAP	Positive airway pressure
RCTs	Randomized controlled trials
SBP	Systolic blood pressure
SGLT2i	Sodium–glucose cotransporter-2 inhibitor
T2DM	Type 2 diabetes mellitus
UACR	Urinary albumin-to-creatinine ratio
